# AL355338 acts as an oncogenic lncRNA by interacting with protein ENO1 to regulate EGFR/AKT pathway in NSCLC

**DOI:** 10.1186/s12935-021-02232-z

**Published:** 2021-10-09

**Authors:** Qian Hua, Dongliang Wang, Lin Zhao, Zhihui Hong, Kairu Ni, Yizhen Shi, Zengli Liu, Baoming Mi

**Affiliations:** 1 Department of Nuclear Medicine, The Second Affiliated Hospital of Soochow University, Suzhou, 215004 Jiangsu China; 2 State Key Laboratory of Radiation Medicine and Protection, School of Radiation Medicine and Protection, Collaborative Innovation Center of Radiation Medicine of Jiangsu Higher Education Institutions, Soochow University, Suzhou, China; 3 Department of Nuclear Medicine, Fudan University Shanghai Cancer Center, Fudan University, Shanghai, 200032 China

**Keywords:** Long noncoding RNAs, Aerobic glycolysis, AL355338, ENO1, EGFR

## Abstract

**Background:**

Non-small cell lung cancer (NSCLC) is a malignancy with considerable morbidity and mortality. Abnormal metabolism is a hallmark of cancer; however, the mechanism of glycolysis regulation in NSCLC progression is not completely understood. Recent studies suggest that some dysregulated long non-coding RNAs (lncRNAs) play important roles in tumor metabolic reprogramming.

**Methods:**

To identify glycolysis-associated-lncRNAs in NSCLC, we compared RNA-sequencing results between high ^18^F-fluorodeoxyglucose (FDG)-uptake NSCLC tissues and paired paratumor tissues. The transcript abundance of AL355338 in 80 pairs of clinical samples was evaluated by quantitative real-time PCR assay and fluorescence in situ hybridization. The biological role of AL355338 on NSCLC cells were evaluated by functional experiments in vitro and in vivo. Moreover, RNA pull-down, mass spectrometry and RNA immunoprecipitation (RIP) assays were used to identify the protein interacted with AL355338. Co-immunoprecipitation, in situ proximity ligation assays and western blotting were applied to define the potential downstream pathways of AL355338.

**Results:**

AL355338 was an upregulated glycolysis-associated lncRNA in NSCLC. Functional assays revealed that AL355338 was critical for promoting aerobic glycolysis and NSCLC progression. Mechanistic investigations showed that AL355338 directly bound with alpha-enolase (ENO1) and enhanced the protein’s stability by modulating its degradation and ubiquitination. A positive correlation was observed between AL355338 and ENO1 in NSCLC, and ENO1 was subsequently confirmed to be responsible for the oncogenic role of AL355338. Furthermore, AL355338 was capable of modulating ENO1/EGFR complex interaction and further activating EGFR-AKT signaling.

**Conclusions:**

This study indicates that AL355338 confers an aggressive phenotype to NSCLC, and targeting it might be an effective therapeutic strategy.

**Supplementary Information:**

The online version contains supplementary material available at 10.1186/s12935-021-02232-z.

## Background

Among malignancies, lung cancer is one of the most lethal with high global mortality and incidence rates [1]. Non-small cell lung cancer (NSCLC) is the most commonly diagnosed type, accounting for ~ 85% of cases [2]. Early diagnosis of NSCLC is difficult, and most patients are diagnosed at advanced stages and have poor clinical outcomes [2]. In-depth investigation into the molecular alterations and their mechanisms that underlie NSCLC progression is extremely essential to develop and optimize therapeutic strategies.

Cancer cell growth depends on sufficient energy. Previous studies suggested that glucose metabolism reprogramming, also known as the Warburg effect, provided fuel for cancer’s malignant behavior [3]. ^18^F-fluorodeoxyglucose (FDG) is an analogue of glucose and radioactive tracer that enables tracking of glucose uptake in solid tumors in positron emission tomography/computed tomography (PET/CT) imaging, which is the gold standard for quantifying glycolytic metabolism [4]. Enhanced glucose uptake ability has been observed in patients with various types of cancer, and especially in NSCLC [5]. Cancer biology studies using genomic techniques revealed that the orchestrated performance of oncogenes and downstream pathway activation exacerbate metabolic disorder [6]. However, the underlying mechanistic details underlying glycolysis regulation in NSCLC progression remain to be determined.

Long non-coding RNAs (lncRNAs) are RNA transcripts (> 200 nucleotides) with limited coding potential [7] that are abnormally expressed in all stages of cancer development and play vital regulatory roles in oncogenic processes [8]. A recent study reported that lncRNA dysregulation is closely related to tumor metabolic reprogramming [9]. Further work is needed to identify glycolysis-related-lncRNAs in NSCLC and clarify their potential roles and specific mechanisms.

Alpha-enolase (ENO1) is an enolase isoform originally characterized as a crucial glycolytic enzyme catalyzing the dehydration of 2-phosphoglycerate into phosphoenolpyruvate; it also generates ATP during glycolysis [10]. Besides its well-known enzymatic functions, decades of research have shown that ENO1 is aberrantly expressed in multiple tumor types and participates in diverse cellular processes through non-metabolic mechanisms [11]. Interestingly, some studies reported that lncRNAs interact with ENO1 and affect its expression or activity [12, 13], highlighting a novel approach to elucidate the non-metabolic roles of metabolic genes in cancer.

Recently, we aimed to identify glycolysis-associated-lncRNAs through RNA-sequencing (RNA-seq) of high ^18^F-FDG-uptake NSCLC tissues and paired paratumor tissues. AL355338 was the top-scoring upregulated lncRNA. In this present study, we observed that high AL355338 expression positively correlated with NSCLC clinical stage and worse patient prognosis. Functional assays demonstrated the influences of AL355338 silencing on NSCLC proliferation, metastasis, and glycolysis in vitro and in vivo. Mechanistically, our evidence indicated that AL355338 could directly interact with ENO1 and stabilize the protein, preventing its ubiquitination and degradation. AL355338 exerted its biological functions partially by upregulating ENO1 expression. Further experiments showed that AL355338 positively modulated the interaction between ENO1 and epidermal growth factor receptor (EGFR), subsequently activating EGFR/AKT signaling. Collectively, these results strongly suggest that the AL355338-ENO1-EGFR/AKT regulatory axis may harbor NSCLC diagnostic biomarkers and therapeutic targets.

## Materials and methods

### Patient and clinical information

Eighty paired NSCLC tissues and paratumor tissues were collected from the surgery specimen archives of the Second Affiliated Hospital of Soochow University. Patient follow-up continued to December 2020. No recruited patients received preoperative chemotherapy or radiotherapy. This research was approved by the institutional clinical research ethics committee of the Second Affiliated Hospital of Soochow University. Patient stratification was based on the new TNM staging criteria in lung cancer (8th edition) [14].

### Cell culture and treatment

The human normal lung epithelial cell line HBE and four human NSCLC cell lines (A549, PC9, H1650, H1299) were obtained from ATCC (Manassas, VA, UA) and cultured in Dulbecco's modified Eagle’s medium (Gibco, Grand Island, NY, USA) supplemented with 10% fetal bovine serum (Gibco) at 37 °C, in a humid 5% CO_2_ incubator. Two small interfering RNA (siRNA) constructs (GenePharma, Shanghai, China) were designed to knock down AL355338 expression, and non-specific siRNA served as the negative control; all siRNAs were transfected with Lipofectamine 2000 (Invitrogen, Carlsbad, CA, USA). For ectopic AL355338, ENO1, and EGFR expression, the appropriate cDNA was amplified with reverse transcription polymerase chain reaction (RT-PCR) and subcloned into a pcDNA-4/TO vector. Neofect transfection reagent (Neofect biotech, Beijing, China) was used for plasmid transfection. The sh-AL355338, sh-ENO1 and sh-NC lentiviruses were purchased from GenePharma. The siRNA and sh-RNA sequences are listed in Additional file 1: Table S1.

### Total RNA isolation and quantitative RT-PCR (qRT-PCR) assays

Total RNA was extracted from cells or tissues with TRIzol reagent (Omega, Norcross, GA, USA) according to the manufacturer’s instructions. RNA quality and quantity were assessed with the NanoDrop2000 platform (Thermo Fisher Scientific, Waltham, MA, USA). cDNA Synthesis kits (TaKaRa, Otsu, Japan) were used for mRNA reverse transcription. Real-time PCR analysis was performed with SYBR Green PCR Kits (TaKaRa) on the 7500 Sequence Detection System (Applied Biosystems, Foster City, CA, USA). The amplified transcript levels of genes were normalized to β-actin with the optimized comparative 2^−ΔΔCt^ value method. The primer sequences are in Additional file 1: Table S1.

### Nuclear-cytoplasmic fractionation

The nuclear and cytoplasmic fractions were generated with a PARIS™ Kit (Ambion, Austin, TX, USA) according to the manufacturer protocol. Nuclear and cytoplasmic RNA levels were normalized to U6 and β-actin, respectively using real-time PCR.

### Fluorescence in situ hybridization (FISH)

FISH analyses were performed with RNA FISH kits (RiboBio, Guangzhou, China) using a previously described method [15]. The AL35538 probe sequence was 5′-DIG-ACTTCGAGACCAGCATGGCCAACATGGTGAAGCCC-DIG-3′. Nuclei were stained with DAPI, and high-resolution images were taken with a confocal microscope (ZEISS, Oberkochen, Germany). Percentages of positive stained cells were quantified as: 1 = 0–25%, 2 = 26–50%, 3 = 51–75%, and 4 = 76–100%. Staining intensity scores were: 0 = none, 1 = weak, 2 = moderate, and 3 = strong. The final scores were calculated by multiplying the percentage and intensity scores of positive cells. Samples were divided into low (score 0–6) and high (score 7–12) expression groups.

### Flow cytometry assay

Transfected cells were harvested and fixed in pre-cooled 70% ethanol for 1 h on ice. Then cells were washed with cold phosphate-buffered saline (PBS) three times and incubated with RNase for 30 min at 37 °C. After staining the cells with propidium Iodide for 15 min at room temperature, they were analyzed with a flow cytometer (BD Biosciences, Franklin Lakes, NJ, USA). The percentages of cells in the G0/G1, S, and G2/M phases were calculated using ModFit software (Verity Software, Topsham, ME, USA).

### Cell proliferation assay

Cell proliferation rates were measured with Cell Counting Kit-8 (bimake, Shanghai, China) according to the manufacturer’s protocol. Briefly, 5 × 10^3^ cells per well were seeded into 96-well plates. At 0, 24, 48, and 72 h after cell transfection, 10 μL CCK-8 solution was added to each well. After incubating he plates for 2 h at 37 °C, the optical density at 450 nm was measured for each well. For colony formation assays, transfected or control A549 and PC9 cells were harvested 24 h after transfection, and 500 cells per well were seeded into 6-well plates. After 2 weeks of incubation, the colonies were fixed in 4% paraformaldehyde and stained with crystal violet solution. Cell proliferation was also assessed using ethynyldeoxyuridine (EdU) analyses (RiboBio) according to the manufacturer’s instructions as previously reported [16].

### Cell migration and invasion assay

Wound healing assays were carried out as previously described [17]. Transwell assays were performed using Boyden chambers containing transwell membrane filters (Corning, Corning, NY, USA). The detailed protocol was published elsewhere [17]. Images were collected with an inverted microscope (Olympus, Tokyo, Japan) and analyzed with ImageJ software (National Institutes of Health, Bethesda, MD, USA). At least five random fields of view were analyzed in each chamber.

### Measurement of glucose uptake, lactate production, and ATP

As a glucose analogue, ^18^F-FDG uptake reflects the intracellular glucose uptake level. In vitro experiments were performed as previously reported [16]. Lactate production was measured in cells that were seeded into 12-well plates in triplicate for 24 h and then refreshed with 1 mM glucose culture medium overnight. Culture supernatants were collected to measure lactate concentrations (Nanjing Jiancheng Bioengineering Institute, Nanjing, China), and lysed cell pellets were used to measure ATP levels (Nanjing Jiancheng) using the manufacturer’s instructions. ^18^F-FDG uptake and lactate production were normalized to number of cells in each sample; ATP levels were normalized to cell protein mass.

### Western blot

Western blotting was performed following a standard protocol [18]. The antibodies used are listed in Additional file 1: Table S2. Immunoreactive bands were detected using ECL western blot kits (Amersham Biosciences, Little Chalfont, UK).

### Immunofluorescence (IF)

IF was performed as described elsewhere [18]. Protein expression and localization were observed under a confocal microscope (ZEISS). Antibodies used for IF are listed in Additional file 1: Table S2.

### RNA pull-down assay

Biotin-RNA pull-down assays were performed as described elsewhere [15]. Briefly, full-length sense, antisense, and serial deletion sequences of AL355338 were amplified with a T7-containing primer and reverse transcribed using MAXIscript™ T7 Transcription Kits (Thermo Fisher Scientific). Pierce™ RNA 3′ End Desthiobiotinylation Kits (Thermo Fisher Scientific) were used to label targeted RNA. The biotin-labeled AL355338 probe was incubated with total cell lysates of A549 cells using Pierce™ Magnetic RNA–Protein Pull-Down Kits (Thermo Fisher Scientific). Eluted proteins were purified by silver staining and subjected to mass spectrometry (MS) or western blotting.

### RNA immunoprecipitation (RIP) assay

Magna RNA-binding protein immunoprecipitation kits (Millipore, Billerica, MA, USA) were used to determine specific protein binding with targeted RNA according to the manufacturer’s protocol and as described previously [15]. Briefly, cells were lysed with RIP lysis buffer, and lysates were incubated overnight at 4 °C with magnetic beads conjugated with human anti-ENO1 antibody or anti-mouse immunoglobulin G (IgG). The next day, co-immunoprecipitated RNA was extracted for qRT-PCR analyses. Total RNA (input controls) and IgG controls were simultaneously assayed to confirm that the RNA specifically bound to ENO1.

### Co-immunoprecipitation (Co-IP)

Reciprocal exogenous and endogenous co-IP assays were performed as described elsewhere reported [18]. IP samples and input controls were analyzed by western blot. The co-IP antibodies are listed in Additional file 1: Table S2.

### Proximity ligation assay (PLA)

PLA (Duolink®, Sigma-Aldrich, St. Louis, MO, USA) was performed to evaluate interactions between ENO1 and EGFR as previously reported [19]. Briefly, A549 and PC9 cells were grown on glass coverslips in 24-well plates at 37 °C overnight. The next day, cells were fixed in 4% paraformaldehyde for 20 min and permeabilized with 0.1% Triton X-100 for 5 min. After blocking for 30 min, the cells were incubated with anti-ENO1 and anti-EGFR antibodies diluted in blocking buffer at 4 °C overnight. The next day, cells were incubated with species-specific PLA probes at 37 °C for 1 h, then 1× ligase for 30 min, and finally 1× polymerase diluted with amplification stock solution at 37 °C for 100 min to amplify the fluorescence signal. Finally, coverslips were mounted on slides with anti-fluorescence quenching sealing liquid containing DAPI. Images were acquired under confocal microscopy (ZEISS).

### In vivo xenograft assays

Four-week-old male BALB/c nude mice were purchased from the Animal Center of Soochow University. Animal experiments were carried out according to the protocol approved by the Animal Care and Ethics Committee of the Second Affiliated Hospital of Soochow University. A total of 20 mice were subcutaneously injected with 5 × 10^6^ A549 cells in the right flank with sh-AL355339, and sh-NC was given in the left flank to establish an NSCLC xenograft model. Tumor volume was measured every 5th day and calculated according to the equation V = length × width^2^ × 1/2. Micro-PET/CT (Super Nova® PET/CT, Pingseng, China) was performed 3 weeks after cell injection to measure ^18^F-FDG uptake by xenograft tumors. All tumor-bearing mice were fasted for 8 h prior to PET/CT. ^18^F-FDG (0.1 mL, 7.4 MBq) was injected via tail vein. After 30 min, they were anesthetized with 2% isoflurane in oxygen and immobilized for 15-min scans. ^18^F-FDG uptake was measured as maximum standard uptake (SUV_max_) in the regions of interest covering the tumors. After 3 weeks, mice were humanely sacrificed by euthanasia. The mice were inhaled with CO_2_ for 5 min. The CO_2_ was gradually filled into an airtight chamber at 30% of the chamber volume/min rate. The xenograft tumors were harvested and measured.

### In vivo metastasis assays

Stably transfected A549 cells (1 × 10^6^/0.2 ml PBS) were injected into the tail vein of nude mice. Micro-CT scans were performed 5 weeks later to observe lung metastasis. After 5 weeks, mice were humanely sacrificed by euthanasia. Following sacrifice, the lungs were surgically dissected and embedded in paraffin for hematoxylin and eosin (HE) staining. The numbers of pulmonary metastatic nodules were compared between groups.

### Immunohistochemistry (IHC)

IHC was performed and quantified as previously described [20].

### Statistical analysis

All data are expressed as mean ± standard deviation. Statistical analysis was performed with SPSS (version 22.0, IBM Corp., Armonk, NY, USA) and graphs were generated with GraphPad Prism software (version 8.0, GraphPad Inc., San Diego, CA, USA). Student’s t-tests or one-way analyses of variance were used to assess significant differences between groups. Categorical data were compared with Chi-square tests. Survival analyses were performed using Kaplan–Meier analyses.

## Results

### AL355338 was upregulated in aggressive NSCLC and associated with poor patient prognosis

To identify glycolysis-related lncRNAs in NSCLC, we performed RNA-seq assay with ^18^F-FDG high-uptake NSCLC tissues and pair-matched normal tissues from our previous study [16]. We detected 222 upregulated genes (fold change > 1.5, false discovery rate < 0.005). The top 20 significantly upregulated lncRNAs (Additional file 1: Figure S1A) were submitted for Gene Ontology analysis, and the results showed that they were most enriched in metabolic pathways (Additional file 1: Figure S1B). To screen more functional lncRNAs, our next investigation used more stringent filtering criteria (> 400 bp, not fully overlapped with other coding transcripts, efficient PCR amplification). In the current study, we focused on the uncharacterized AL355338, which was the top-scoring upregulated lncRNA in the RNA-seq data, and explored its function in NSCLC development. AL355338 is a 913-bp transcript on human chromosome 13 with 2 exons (Additional file 1: Figure S1C). The Coding-Potential Assessment Tool (CPAT), PhyloCSF tool, and ORFfinder all predicted that AL355338 had no coding potential (Additional file 1: Figure S1D, E). To explore the clinical significance of AL355338, we analyzed RNA-seq data from the publicly available TCGA dataset (StarBase V3.0 platform). AL355338 expression was significantly upregulated in lung adenocarcinoma and lung squamous carcinoma tissues (Additional file 1: Figure S2A, B). Kaplan–Meier survival analysis revealed that patients with high AL355338 expression has shorter overall survival than patients with low AL355338 expression (Additional file 1: Figure S2C, D).

**Fig. 1:**
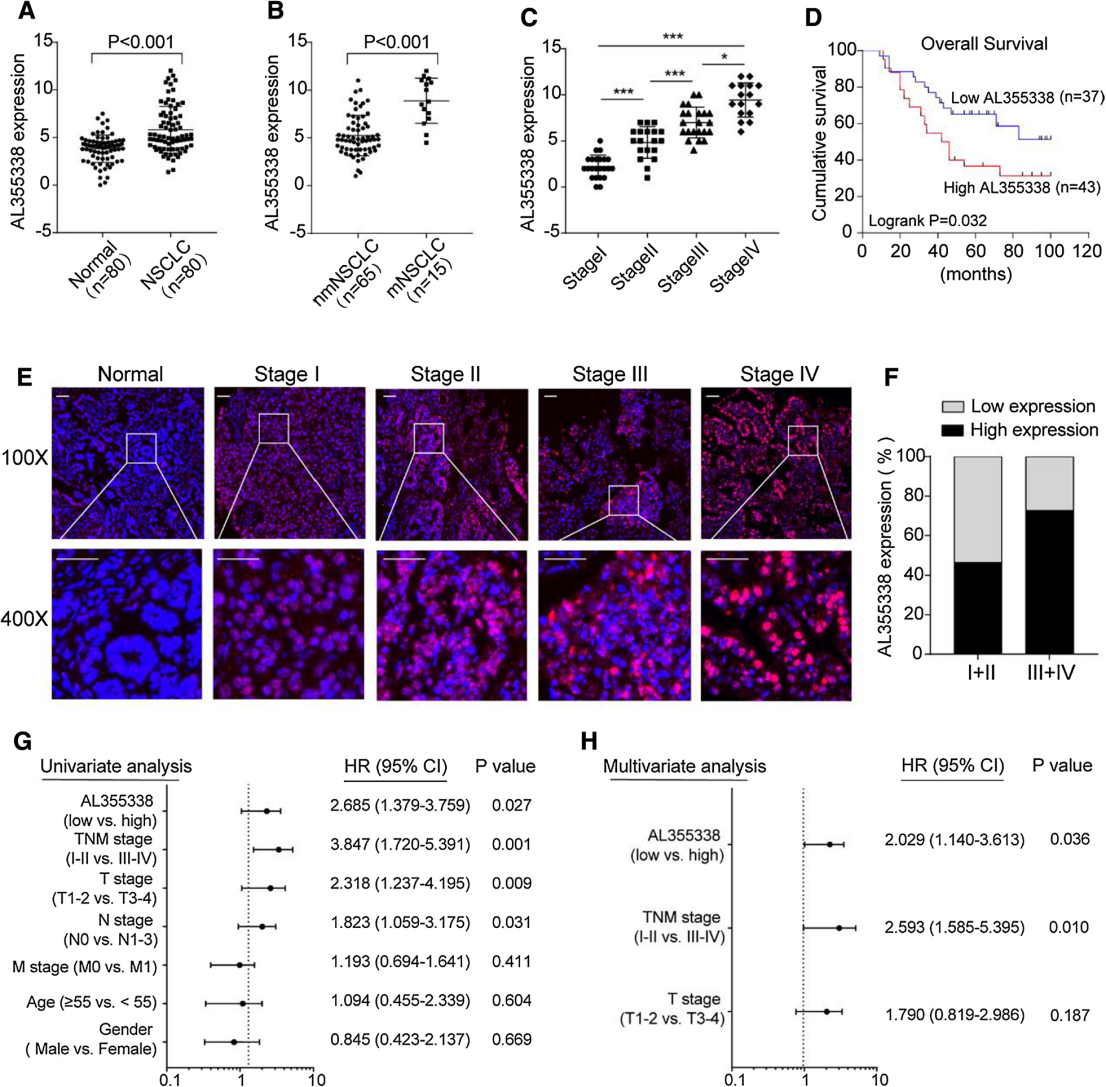
AL355338 was upregulated in aggressive NSCLC tissues and predicted poor prognosis. **A** AL355338 expression was analyzed by qRT-PCR in NSCLC samples and adjacent non-tumor tissues (n = 80). **B** AL355338 expression in metastatic (mNSCLC) samples (n = 65) and non-metastatic (nmNSCLC) samples (n = 15) was measured by qRT-PCR. **C** Relative AL355338 expression levels in different TNM stages. **D** Survival was analyzed and compared between patients with low and high levels of AL355338 in 80 NSCLC patients, log-rank test. **E** Representative FISH images of AL355338 expression in NSCLC tumor tissues and in different TNM stages (blue, DAPI; red, positive staining). Scale bar, 100 μm. **F** Statistical analysis of AL355338 expression in stage I + II group and in stage III + IV group. **G**, **H** Univariate analysis (**G**) and multivariate analysis (**H**) of AL355338 were performed in our NSCLC cohort. All the bars correspond to 95% confidence intervals. Data shown are mean ± SD (n = 3) (*P < 0.05, **P < 0.01, ***P < 0.001)

QRT-PCR analysis confirmed that AL355338 was increased in tumor tissues compared with the corresponding paratumor tissues in a panel of 80 specimens from NSCLC patients (Fig. 1A). Furthermore, AL355338 expression was remarkably higher in metastatic samples compared with non-metastatic samples, and elevated AL355338 expression correlated with advanced TNM stage (Fig. 1B, C). Kaplan–Meier survival analysis of overall survival showed that high AL355338 expression was associated with shorter survival (Fig. 1D; P = 0.032). We next detected AL355338 with FISH and evaluated the relationship between its expression and clinicopathological characteristics. Consistent with the qRT-PCR results, more positive AL355338 expression signals were observed in tumor tissues compared to adjacent normal tissues (Fig. 1E). Expression score analyses demonstrated that expression percentages of AL355338 were higher in stage III + IV than in stage I + II NSCLC samples (Fig. 1F). High AL355338 expression was positively associated with T stage, N stage, distant metastasis, and TNM stage (Table 1). However, it was not related to patient age, sex, or tumor location. Uni- and multivariate regression analyses revealed that AL355338 expression was an independent predictor of NSCLC aggressiveness and could predict clinical outcome (Fig. 1G, H). Collectively, these results suggested that AL355338 was upregulated in NSCLC and might be a useful prognostic biomarker.

**Table 1:** Correlation of the expression of AL355338 in NSCLC with clinicopathologic features

Characteristics		AL355338		P value
		Low (n)	High (n)	
Age (years)				
< 60	34 (42.5%)	16	18	0.54
≥ 60	46 (57.5%)	21	25	
Gender				
Male	41 (51.2%)	17	24	0.26
Female	39 (48.8%)	20	19	
TNM stage				
I	23 (28.7%)	19	4	< 0.01**
II	19 (23.8%)	14	5	
III	22 (27.5%)	4	18	
IV	16 (20.0%)	0	16	
T stage				
T1	21 (26.3%)	12	9	0.04*
T2	37 (46.3%)	19	18	
T3	16 (20.0%)	5	11	
T4	6 (7.5%)	1	5	
N stage				
N0	32 (40.0%)	23	9	0.03*
N1	17 (21.3%)	7	10	
N2	23 (28.7%)	6	17	
N3	8 (10.0%)	1	7	
M stage				
M0	65 (81.3%)	37	28	< 0.01**
M1	15 (18.8%)	0	15	
Mortality				
Survive	30 (37.5%)	17	13	0.04*
Die	50 (62.5%)	20	30	
Histological grade				
I	5 (6.2%)	2	3	0.38
II	42 (52.5%)	24	18	
III	33 (41.3%)	11	22	
Tumor location				
Left	35 (43.8%)	15	20	0.55
Right	45 (56.2%)	22	23	

*P < 0.05; **P < 0.01

### AL355338 promoted NSCLC cell proliferation in vitro

**Fig. 2:**
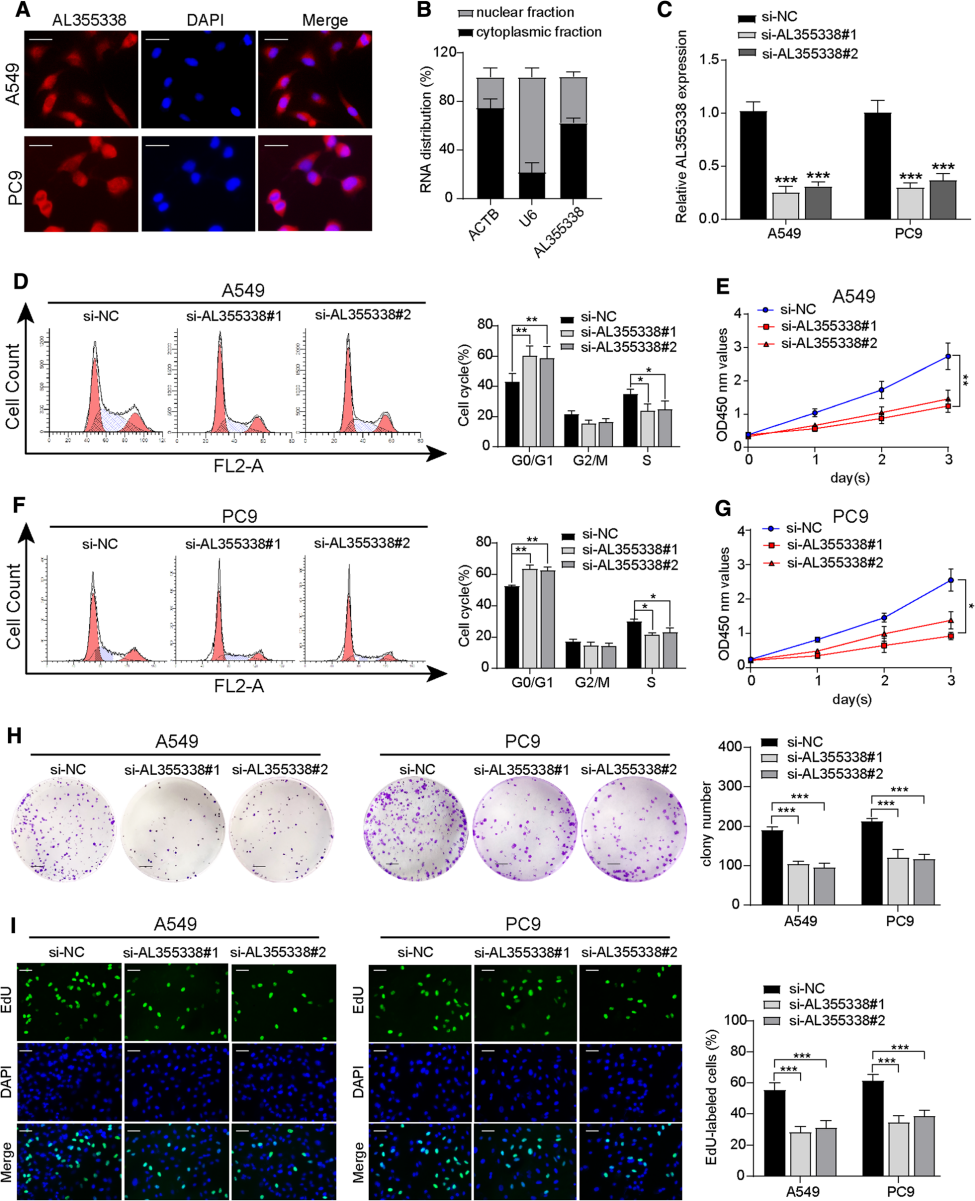
AL355338 promoted NSCLC cell proliferation in vitro. **A** Representative FISH images showed the sub-cellular location of AL355338 in A549 and PC9 cells (blue, DAPI; red, positive staining). Scale bar, 20 μm. **B** Expression of AL355338 in cytoplasmic and nuclear fractionations of A549 cells. **C** The knockdown efficiency of AL355338 in A549 and PC9 cells after being transfected with two siRNAs (si-AL355338#1 and si-AL355338#2). **D**, **F** Relative cell cycle distribution was evaluated by flow cytometry assays in A549 and PC9 transfected with AL355338 siRNAs or negative control (si-NC). **E**, **G** Cell proliferation was performed by CCK-8 assay over a 3-day period in A549 and PC9 cells transfected with AL355338 siRNAs or si-NC. **H** Cell colony formation assay was used to assess the ability of cell proliferation in indicated cells. Scale bar, 100 μm. **I** Immunofluorescence analysis of EdU was performed in AL355338 knockdown cells after 24 h. Scale bar, 50 μm. Data shown are mean ± SD (n = 3) (*P < 0.05, **P < 0.01, ***P < 0.001)

To explore the potential function of AL355338 on cellular behavior, we first detected the expression levels of AL355338 in NSCLC cell lines. Its expression in NSCLC cell lines was significantly higher than that in the normal lung epithelial cell line HBE (Additional file 1: Figure S3A). We then examined its subcellular localization in NSCLC cells. Cellular FISH experiments and analyses of RNA isolated from the cytoplasmic and nuclear compartments of NSCLC cells showed that AL355338 was mainly present in the cytoplasm (Fig. 2A, B). Then, we downregulated AL355338 by transfecting two different siRNAs in A549 and PC9 cells. qRT-PCR was performed to verify the knockdown efficiency of AL355338; both si-AL355338#1 and si-AL355338#2 successfully downregulated AL355338 expression to 20–30% in A549 and PC9 cells (Fig. 2C).

Flow cytometry analyses of cell cycle distribution indicated that AL355338 downregulation significantly decreased the number of cells in S phase, with an increase of cells in G0/G1 phase for both A549 and PC9 cells (Fig. 2D–F). CCK-8 and cell colony formation assays demonstrated that the proliferation and colony formation ability of A549 and PC9 cells were impaired after AL355338 knockdown (Fig. 2E, G, H). Subsequent EdU assays indicated that DNA synthesis by A549 and PC9 cells was markedly reduced after silencing AL355338 (Fig. 2I). These results indicated that AL355338 was effective in facilitating NSCLC cell proliferation.

### AL355338 facilitated aggressive phenotypes and glycolytic metabolism of NSCLC cells

**Fig. 3:**
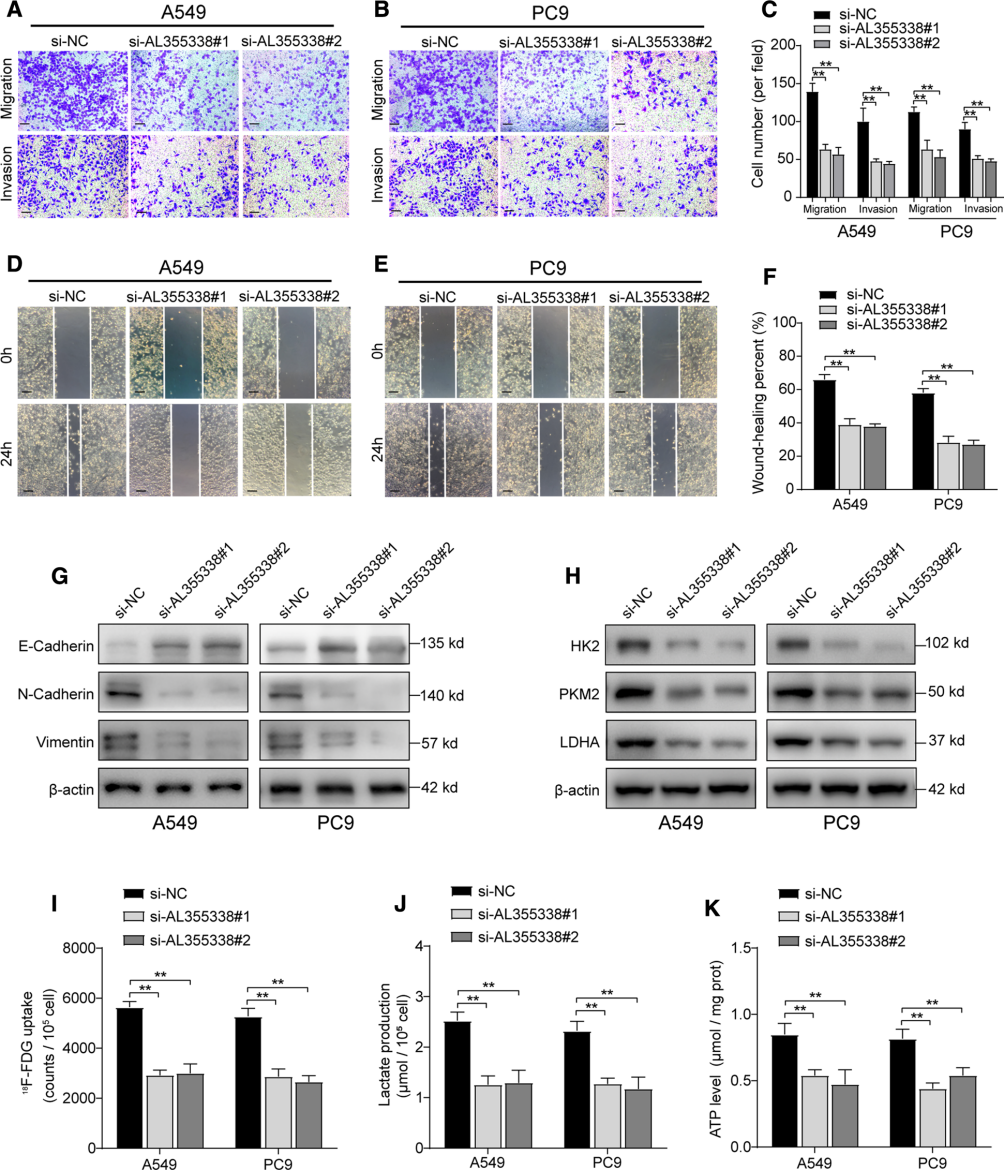
AL355338 facilitated aggressive phenotypes and glycolytic metabolism of NSCLC cells. **A**–**C** The migratory and invasive activities of AL355338 was detected by transwell assays in A549 and PC9 cells transfected with AL355338 siRNAs or si-NC. Representative images and quantitative analysis were shown. Scale bar, 50 μm. **D**–**F** Representative images and quantitative analysis of wound healing assays for the indicated cells. Scale bar, 100 μm. **G** Western blot analysis was performed to detect EMT markers (E-cadherin, N-cadherin, Vimentin) in indicated cells. **H** HK2, PKM2 and LDHA protein expressions were examined under AL355338 knockdown conditions by western blotting. **I** ^18^F-FDG uptake level was detected in A549 and PC9 cells transfected with AL355338 siRNAs or si-NC. **J** Lactate release level was measured for the indicated cells. **K** ATP production level was determined for the indicated cells. Data shown are mean ± SD (n = 3) (*P < 0.05, **P < 0.01, ***P < 0.001)

To further assess the migratory and invasive ability conferred by AL355338, we performed transwell and wound healing assays. The transwell results in Fig. 3A–C show that downregulation of AL355338 suppressed the migratory and invasive activities of A549 and PC9 cells compared to the negative control. Wound healing assays showed the same pattern (Fig. 3D–F). The initiation of epithelial-mesenchymal transition is a driving force of tumor invasion and metastasis. Analyses of the protein expression of epithelial and mesenchymal markers revealed that AL355338 silencing reduced N-cadherin and vimentin expression but enhanced E-cadherin expression in A549 and PC9 cells (Fig. 3G). Consistently, IF staining also showed that the fluorescence intensity of Vimentin was attenuated in AL355338 downregulated A549 cells (Additional file 1: Figure S3B).

Considering AL355338 expression was upregulated in association with high ^18^F-FDG uptake, we investigated the effect of AL355338 in glycolysis regulation. Glucose enzyme expression levels were examined, and the results showed that alteration of AL355338 expression could positively affect hexokinase II, pyruvate kinase isozymes M2, and lactate dehydrogenase A protein levels (Fig. 3H). To confirm the effect of AL355338 on glycolysis, we measured ^18^F-FDG uptake, lactate release, and ATP production to reflect the flux distribution of glucose. AL355338 knockdown led to a decreases in all three parameters (Fig. 3I–K). These results demonstrated that AL355338 could regulate glycolytic metabolism in NSCLC.

### Silencing AL355338 inhibited tumorigenesis, aerobic glycolysis, and metastasis in vivo

**Fig. 4:**
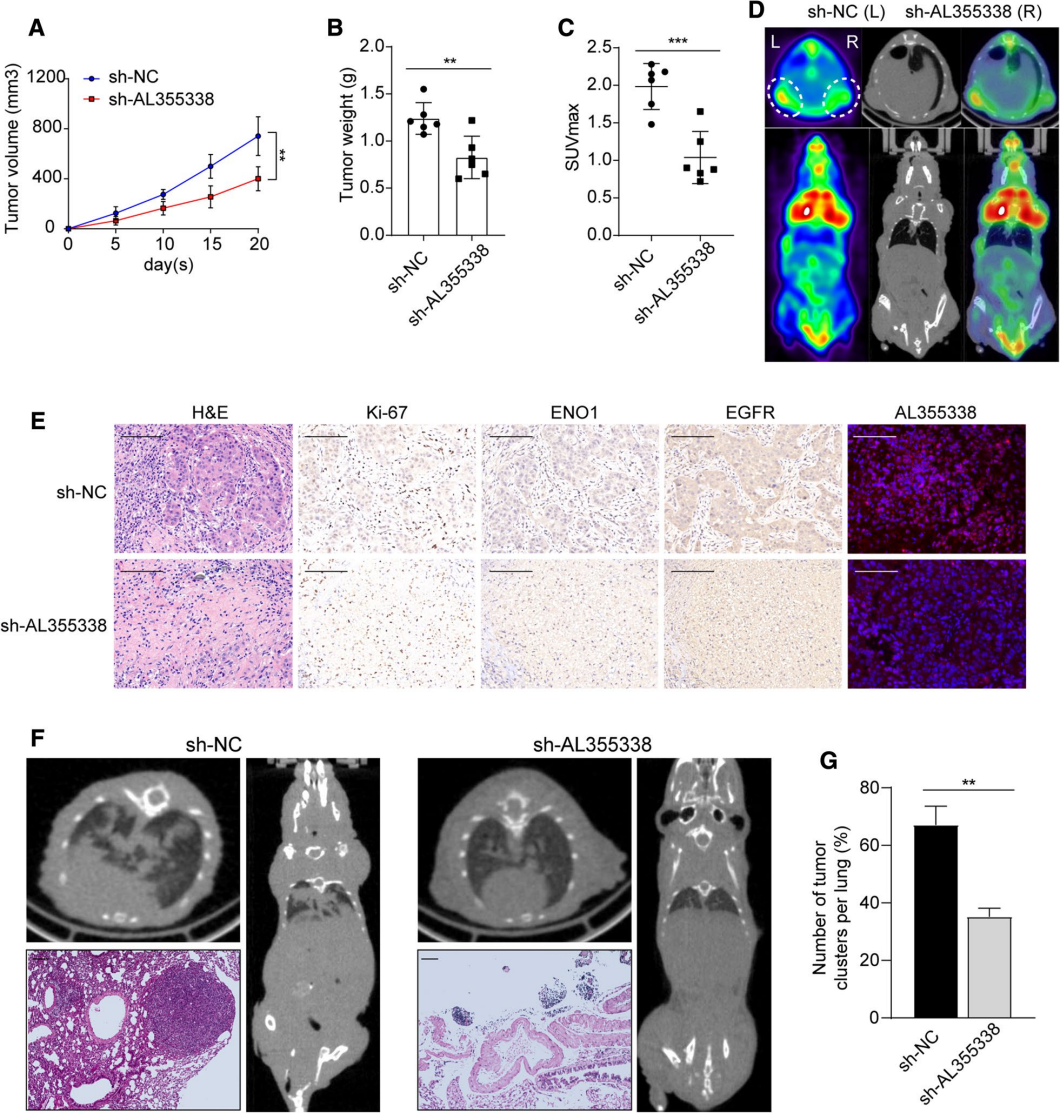
Silencing AL355338 inhibited tumorigenesis, aerobic glycolysis and metastasis in vivo. **A** Tumor volumes were calculated every 5 days until the 3rd week. Tumor growth curves showed that sh-AL355338 group suppressed tumor growth compared with sh-NC group. **B** Tumor weight was measured after harvested from the nude mice with different treatments. **C** ^18^F-FDG micro-PET/CT images of living nude mice were conducted 3 weeks after injection. Comparison of the maximum standard ^18^F-FDG uptake value (SUV_max_) of tumor tissues between the indicated groups. **D** Representative micro-PET/CT images showed higher ^18^F-FDG uptake in sh-NC xenografts compared with sh-AL355338 xenografts. **E** The xenografts were subjected to H&E and IHC staining with Ki-67, ENO1 and EGFR. Representative staining by FISH assay verified AL355338 knockdown efficiency. Scale bar, 100 μm. **F** Tumor cells were injected into nude mice through the tail vein to evaluate the lung homing potential of cells. Representative micro-CT scans images showed the gross lesion in lung tissues and HE staining presented metastatic nodules in the lungs from the different groups. Scale bar, 100 μm. **G** Summarized data on the number of metastatic lung nodules in mice with the indicated groups was counted under HE staining. Data shown are mean ± SD (n = 3) (*P < 0.05, **P < 0.01, ***P < 0.001)

To determine whether AL355338 expression could affect NSCLC cell growth in vivo, A549 cells were stably transfected with sh-AL355338 or negative control sh-NC lentivirus. In vivo xenograft experiments showed that tumor volumes in the sh-AL355338 group were obviously smaller than in the sh-NC group (Fig. 4A). Notably, tumor weights were markedly reduced in the sh-AL355338 group compared with controls (Fig. 4B). In addition, xenograft glycolysis uptake ability was observed by ^18^F-FDG micro-PET/CT imaging. Tumor glycolysis was significantly blocked in sh-AL355338 xenografts, with lower SUV_max_ (mean SUV_max_: 1.15) compared with sh-NC xenografts (mean SUV_max_: 2.08; Fig. 4C, D). IHC revealed that the tumor tissue grown from sh-AL355338 cells displayed lower Ki-67, ENO1, and EGFR staining than those formed from sh-NC cells. FISH analyses confirmed the inhibitory effect of knocking down AL355338 expression in A549 cells (Fig. 4E).

We then investigated the function of AL355338 on tumor metastasis in vivo. Micro-CT images showed more metastatic lesions in the lungs of mice in the sh-NC group, while fewer and smaller metastatic lung nodules were seen in the AL355338 knockdown group (Fig. 4F). Summarized data on HE staining analysis demonstrated that more metastatic lung foci were formed in the sh-NC group compared to the AL355338 knockdown group (Fig. 4G). These results indicated that inhibiting AL355338 expression in NSCLC cells could efficiently suppress tumor metastasis in vivo.

### AL355338 specifically interacted with ENO1 and enhanced ENO1 protein stability

**Fig. 5:**
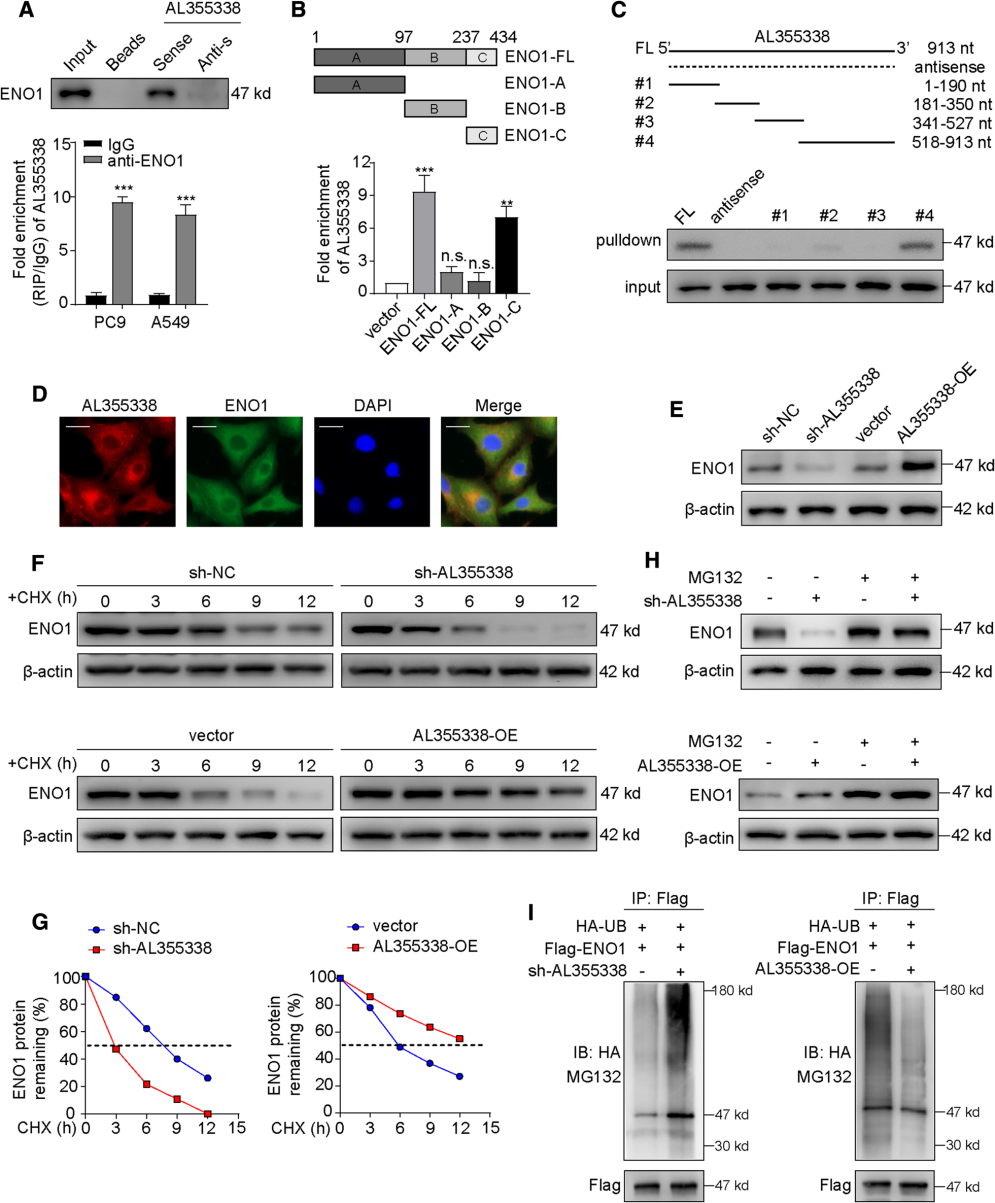
AL355338 specifically interacted with ENO1 and enhanced the stability of ENO1 protein. **A** Proteins retrieved from the AL355338 pull-down assay were analyzed by western blot analysis (in the upper panel). RIP assays using anti-ENO1 antibody showed that ENO1 interacted with AL355338 in A549 and PC9 cells (in the bottom panel). **B** RIP assay was performed in 293T cells transfected with flag-tagged ENO1 vector or its deletion mutants (4/TO-flag vector as negative control). QRT-PCR was used to measure the enrichment of AL355338. **C** Western blot detection of the ENO1 protein in A549 cells as retrieved by in vitro transcribed biotinylated RNAs of different constructs of AL355338 and its antisense sequence serve as negative control. **D** Representative images of co-staining AL355338 (red) and ENO1 (green) in A549 cells by combination of FISH and immunofluorescence. Scale bar, 10 μm. **E** Western blot showed that ENO1 protein level was positively regulated by AL355338 in A549 and PC9 cells. **F** A549 cells transfecting with si-NC or si-AL355338 and PC9 cells transfecting with pcDNA-vector or pcDNA-AL355338 were treated with cycloheximide (CHX, 100 μg/ml) for the indicated periods of time. Cell lysates were analyzed by western blot to examine ENO1 protein half-life. **G** Half-life of ENO1 from CHX-treated protein band intensity was analyzed by ImageJ. **H** A549 cell expressing either si-NC or si-AL355338 and PC9 cell expressing either vector or AL355338-OE were treated with or without MG132 (50 μM) for 6 h. Cell lysates were analyzed by western blot with indicated antibodies. **I** In vitro ubiquitination assay of cells transfected with si-AL355338 in A549 cell (left) or pcDNA-AL355338 in PC9 cell (right). All cells were co-transfected with flag-ENO1 and HA-UB plasmid, 42 h after transfection, cells were incubated with MG132 (50 μM) for 6 h. Cell lysates were immunoprecipitated with anti-flag antibody followed by immunoblotting analysis with anti-HA or anti-flag antibody. Data shown are mean ± SD (n = 3) (*P < 0.05, **P < 0.01, ***P < 0.001)

To investigate the underlying mechanism of AL355338-mediated glycolysis and carcinogenesis in NSCLC, we set out to identify intracellular AL355338-binding factors in an unbiased manner. Biotinylated AL355338 and negative control were incubated with total protein extracts from A549 cells and pulled down with streptavidin (Additional file 1: Figure S4A). MS was conducted following RNA pull-down to screen endogenous AL355338 binding proteins (Additional file 1: Table S3). From the MS data files, we selected ENO1 for further binding validation as it is a key enzyme responsible for the glycolytic pathway. Subsequent western blotting of proteins retrieved from AL355338 pull-down assays confirmed that the ENO1 protein directly bound to the sense AL355338 sequence but did not bind the antisense control (Fig. 5A, upper). In addition, RIP assays with an ENO1 antibody and IgG antibody control were performed using A549 and PC9 cellular extracts. Correspondingly, we observed significantly higher AL355338 enrichment with the ENO1 antibody compared to IgG (Fig. 5A, lower). Both assays demonstrated that AL355338 directly interacted with ENO1. To better identify which region of ENO1 interacted with AL355338, we performed RIP assays using FLAG-tagged truncated forms of ENO1. The results demonstrated that the ENO1-C domain (237–434 aa) was essential for AL355338 binding (Fig. 5B). To map the ENO1-binding regions of AL355338, we constructed four AL355338 deletion fragments. Subsequent RNA pull-down assays followed by western blotting revealed that the ENO1 specific binding sequence was located between 528 and 913 nucleotides (Fig. 5C). RNA FISH of AL355338 combined with IF of ENO1 confirmed cytoplasmic co-localization of AL355338 and ENO1 in A549 cells (Fig. 5D).

A recent study demonstrated that lncRNAs interact with proteins through several modes of action, such as regulating protein expression and function, modulating protein–protein interactions, or guiding protein subcellular localization [21]. To investigate the molecular effect of AL355338 on ENO1, western blotting was performed to measure ENO1 expression after A549 and PC9 cells were subjected to different treatments with AL355338. The results showed that AL355338 positively regulated ENO1 protein expression (Fig. 5E). Similar trends were observed in IF assays (Additional file 1: Figure S4B). Interestingly, loss or gain of function of AL355338 had no influence on ENO1 mRNA expression (Additional file 1: Figure S4C, D), suggesting that AL355338 may regulate ENO1 protein stability. Next, cycloheximide (CHX) chase assays were performed to observe the effect of AL355338 on ENO1 protein degradation. Western blotting showed that ENO1 protein was more stable in AL355338-transfected cells, while it had a shorter half-life in AL355338-silenced cells (Fig. 5F, G). Moreover, the proteasome inhibitor MG132 rescued the reduction of ENO1 caused by AL355338 repression (Fig. 5H), suggesting that AL355338 elevated ENO1 levels by reducing its degradation. As reported, cytoplasmic protein degradation involves the ubiquitin–proteasome pathway [22]. Thus, whether AL355338 was involved in the ubiquitin-mediated ENO1 degradation was tested with in vitro ubiquitination assays. The results illustrated that upregulation of AL355338 decreased ENO1 ubiquitination, whereas silencing of AL355338 enhanced ENO1 ubiquitination (Fig. 5I). Collectively, our data indicated that AL355338 directly bound the ENO1 protein and enhanced its stability in NSCLC cells.

### ENO1 participated in the oncogenic function of AL355338

**Fig. 6:**
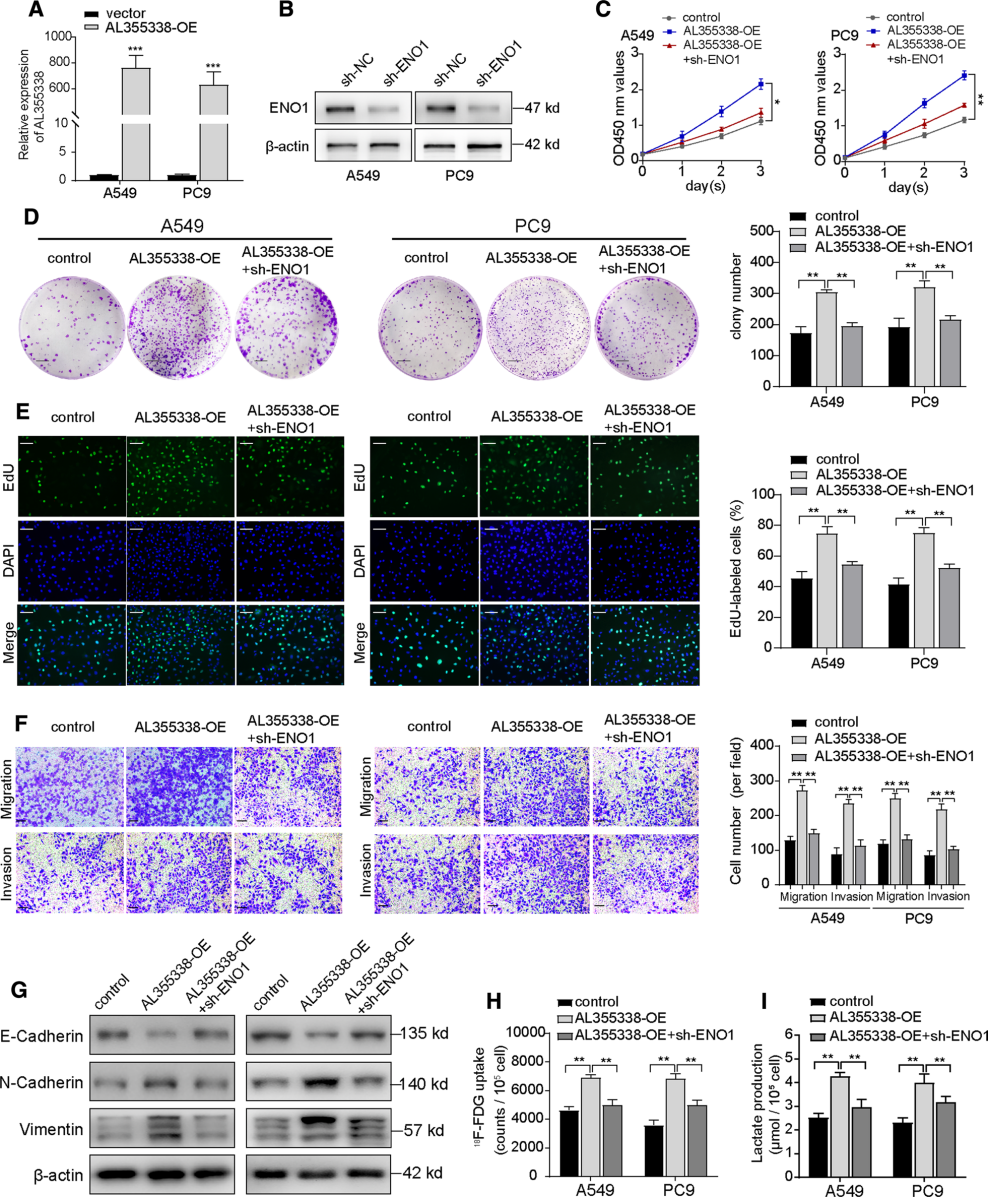
ENO1 participated in the oncogenic function of AL355338. **A** Relative expression levels of AL355338 in A549 and PC9 cells transfected with pcDNA-AL355338. **B** Western blot analysis showed stable downregulation of ENO1 by sh-ENO1 in A549 and PC9 cells. **C**–**E** CCK-8, colony formation and EdU assays demonstrated that knockdown of ENO1 partially attenuated the enhanced cell proliferation induced by overexpression of AL355338 in A549 and PC9 cells. Scale bar = 100 μm (colony) or 50 μm (EdU). **F** Cell migration and invasion were detected by transwell assays to assess the impact of ENO1 silencing on the role of AL355338 overexpression. Scale bar, 50 μm. **G** The effect of AL355338 downregulation on the protein levels of E-cadherin was rescued by ENO1 knockdown, while the upregulation of N-cadherin and Vimentin was reduced. **H**, **I** Metabolic functional rescue experiments showed that ^18^F-FDG uptake and lactate production promoted by ectopic expression of AL355338 could be repressed by sh-ENO1 in A549 and PC9 cells. Data shown are mean ± SD (n = 3) (*P < 0.05, **P < 0.01, ***P < 0.001)

To clarify whether the RNA–protein interaction between AL355338 and ENO1 mediated the key oncogenic function of AL355338 in NSCLC, complementary rescue experiments were performed. Stable ENO1-knockdown A549 and PC9 cells were constructed and then transfected with AL355338 overexpression plasmid. Transfection efficiency was confirmed by qRT-PCR and western blot (Fig. 6A, B). As was expected, CCK-8 and cell colony formation assays showed that ectopic AL355338 expression increased the proliferation activity and colony formation ability of A549 and PC9 cells, while ENO1-knockdown partially abrogated the effects induced by AL355338 upregulation (Fig. 6C, D). EdU assays revealed that increased DNA synthesis capability of A549 and PC9 cells induced with AL355338 was reversed by ENO1 silencing (Fig. 6E). Cell migration and invasion abilities were also assessed, and results demonstrated that AL355338 exerted minimal effects on cell migration and invasion in the absence of ENO1 (Fig. 6F). Importantly, the stimulatory effect of AL355338 on epithelial and mesenchymal marker expression was recovered by repressing ENO1 (Fig. 6G). In glycolysis metabolism assays, ENO1 downregulation dramatically blocked the AL355338-induced increase in ^18^F-FDG uptake, as well as lactate production by A549 and PC9 cells (Fig. 6H, I). The above results suggested that AL355338 regulation of glycolytic metabolism and cell proliferation were dependent on ENO1 stability in NSCLC cells.

### AL355338 activated the EGFR/AKT pathway by targeting the ENO1/EGFR interaction

The EGFR/AKT signaling pathway influences tumor cell survival in many cancers. As previous reports have demonstrated that ENO1 could regulate the AMPK-mediated or FAK-mediated AKT pathway [23, 24], we sought to determine whether ENO1 could interact with EGFR and subsequently activate its downstream AKT kinase signaling pathway. To verify the relationship between ENO1 and EGFR in NSCLC cells, we first examined their TCGA database expression levels. We extracted ENO1 and EGFR data from 34 tumors and found that both were notably upregulated in diverse cancer types, especially lung adenocarcinoma and lung squamous carcinoma tissues (Additional file 1: Figure S5A, B). As shown in Additional file 1: Figure S5C, the correlation coefficient of ENO1 and EGFR reached 0.32 in the NSCLC cohort (P < 0.001, Spearman correlation analysis). Besides, ENO1 shared a similar mutation status with EGFR in NSCLC samples (P < 0.001, Additional file 1: Figure S5D).

**Fig. 7:**
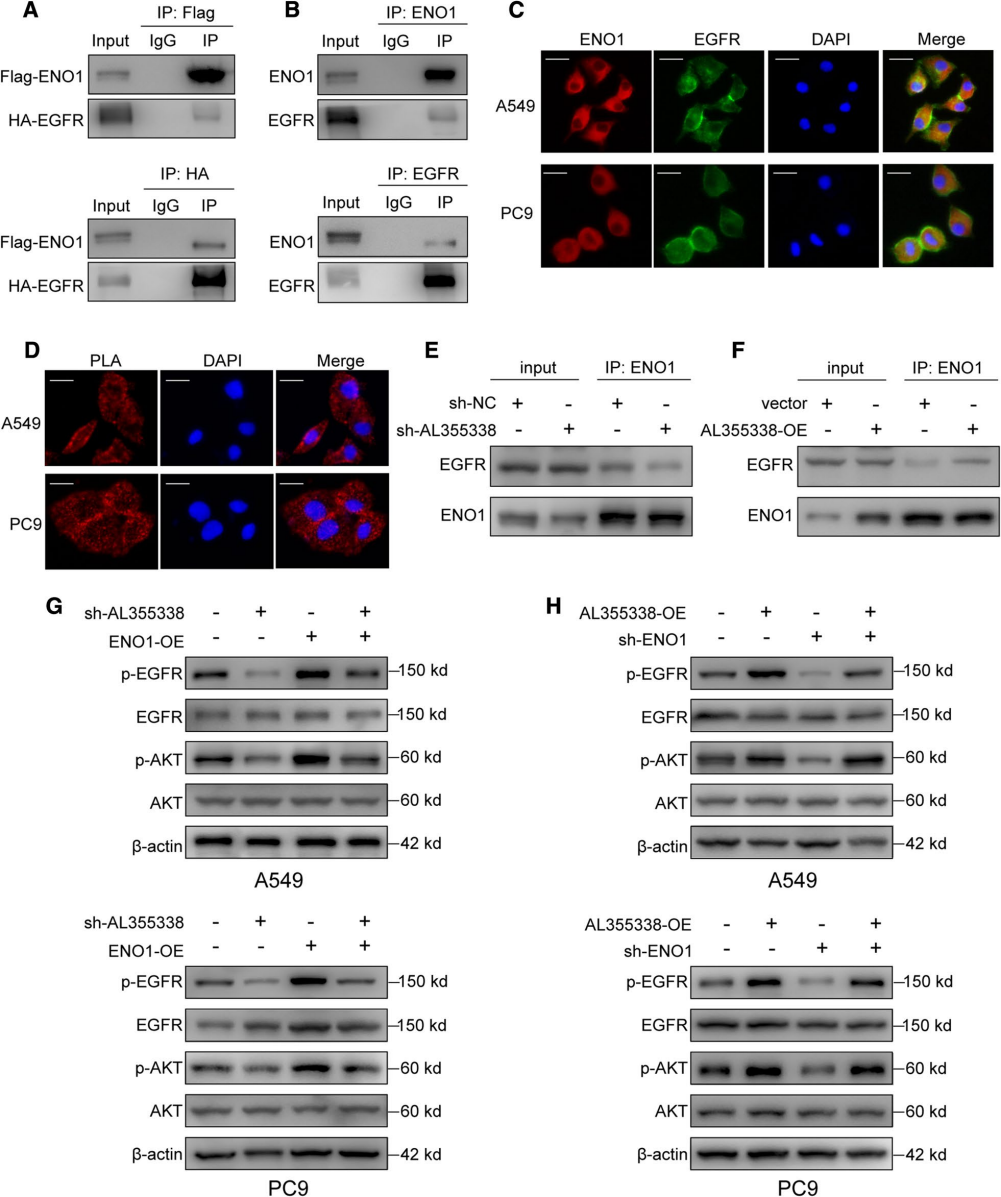
AL355338 activated the EGFR/AKT pathway by targeting ENO1/EGFR interaction. **A** 293T cells were co-transfected with flag-ENO1 and HA-EGFR. Reciprocal immunoprecipitation was performed with anti-HA or anti-flag agarose (IgG as control). Input (whole-cell extracts) and immunoprecipitates were analyzed by SDS-PAGE and immunoblotted with indicated antibodies. **B** Lysates of A549 cells were immunoprecipitated with the antibody against ENO1 or EGFR. Input and immunoprecipitates were analyzed by SDS-PAGE and immunoblotted with indicated antibodies. **C** Subcellular co-localization of ENO1 and EGFR in A549 and PC9 cells. Cells were immunostained with anti-ENO1 (red) and anti-EGFR (green). The nucleus was marked with DAPI (blue). Scale bar, 10 μm. **D** In situ proximity ligation assay (PLA) demonstrated the interaction between ENO1 and EGFR in A549 and PC9 cells. Positive PLA signals demonstrated ENO1/EGFR complex which were shown as red clusters, and cell nucleus were counterstained with DAPI (blue). Scale bar, 10 μm. **E**, **F** The interaction between ENO1/EGFR was detected in AL355338-knockdown A549 cells and AL355338-overexpressing PC9 cells using co-IP assays. **G** Western blotting analysis of p-EGFR, EGFR, p-AKT, AKT in A549 and PC9 cells treated with sh-AL355338 alone or co-transfection with ENO1 overexpression plasmid. **H** Western blotting analysis of p-EGFR, EGFR, p-AKT, AKT in A549 and PC9 cells treated with AL355338 overexpression alone or co-transfection with sh-ENO1

Next, we aimed to investigate whether ENO1 directly interacted with EGFR. Reciprocal co-IP assays were performed using both exogenous labeled antibodies and endogenous antibodies to precipitate ENO1 and EGFR, respectively. The co-IP results confirmed physical binding between ENO1 and EGFR proteins in NSCLC cells (Fig. 7A, B). IF staining confirmed ENO1 and EGFR co-localization in A549 and PC9 cells (Fig. 7C). Furthermore, in situ PLA was used to visualize native protein complexes. High densities of PLA-positive protein complexes that included ENO1 and EGFR were observed in A549 and PC9 cells, which was in accordance with the co-IP results (Fig. 7D). Intriguingly, AL355338 knockdown noticeably impaired the ENO1 and EGFR interaction, while AL355338 overexpression had the opposite effect (Fig. 7E, F).

To understand the potential relationship between AL355338 and the EGFR/AKT pathway, we investigated EGFR and AKT expression. Western blot analyses showed that AL355338 suppression decreased the levels of phosphorylated EGFR and AKT, but total protein levels were not affected. Overexpression of AL355338 enhanced EGFR and AKT phosphorylation, indicating that the EGFR/AKT pathway was a downstream effector of AL355338. We then assessed the ability of ENO1 to overcome AL355338-mediated activation of the EGFR/AKT pathway in NSCLC cells. Notably, ENO1 knockdown abrogated AL355338-mediated increases of EGFR and AKT phosphorylation, whereas ENO1 overexpression reversed sh-AL355338-mediated inactivation of the EGFR/AKT pathway (Fig. 7G, H). Based on these results, we inferred that AL355338 could regulate EGFR/AKT pathway activity by targeting ENO1/EGFR interaction.

### ENO1 was upregulated in NSCLC and positively correlated with AL355338 expression

**Fig. 8:**
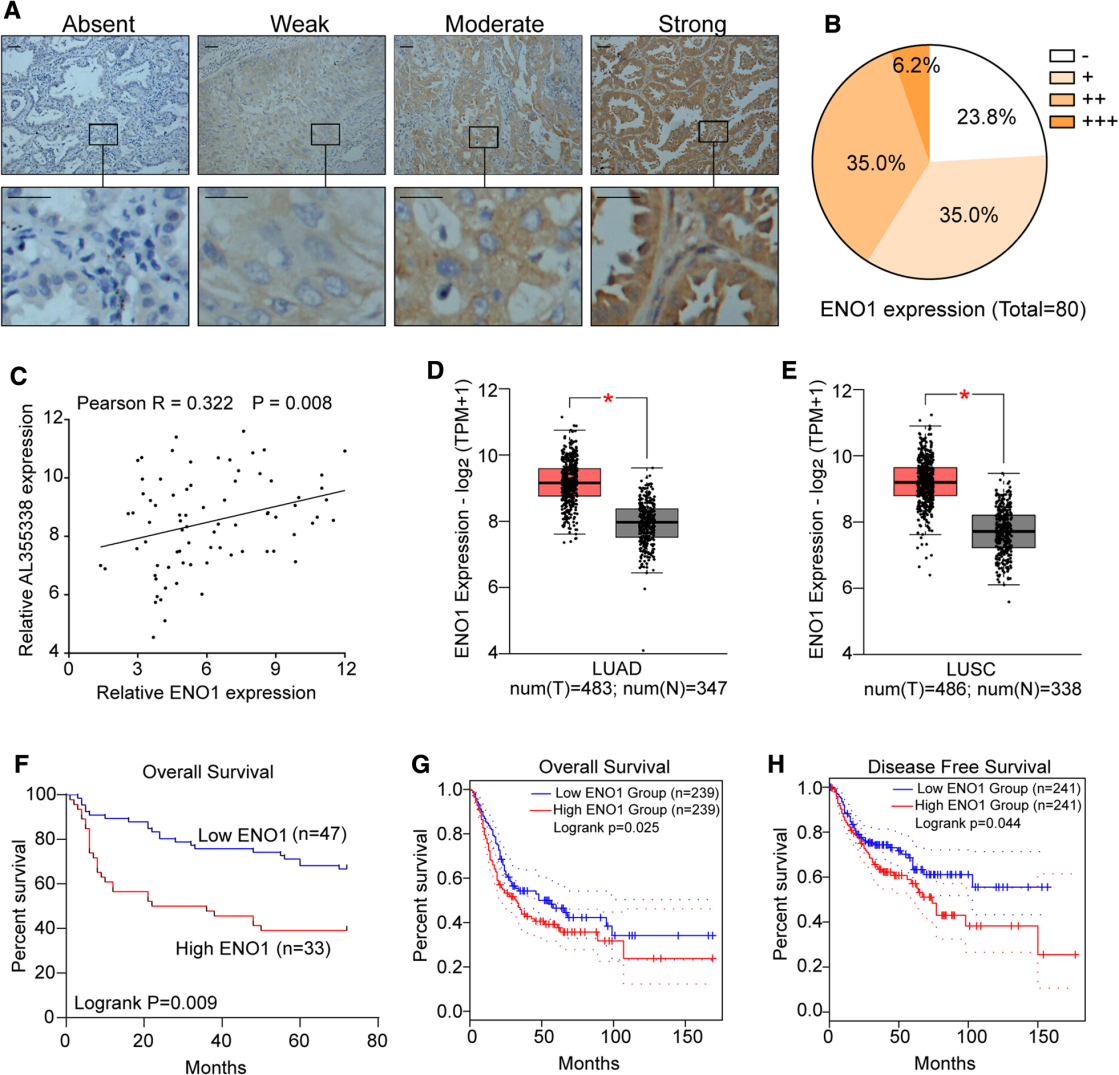
ENO1 was upregulated in NSCLC and positively correlated with AL355338 expression. **A** ENO1 expression was analyzed by IHC in NSCLC specimens from 80 patients. Representative images are shown for absent, weak, moderate and strong expression of ENO1. Scale bar, 100 μm. **B** The distribution of ENO1 expression level in IHC. **C** A positive correlation between ENO1 and AL355338 expression was observed in NSCLC specimens from 80 patients (R = 0.322, P = 0.008 by Spearman correlation test). **D**, **E** The expression pattern of ENO1 in lung adenocarcinoma and lung squamous carcinoma based on TCGA data from GEPIA platform (http://gepia2.cancer-pku.cn). **F** Kaplan–Meier analysis of overall survival of the 80 patients with NSCLC according to different ENO1 levels. **G**, **H** TCGA data from GEPIA Platform further demonstrated that high ENO1 expression indicated poor survival of NSCLC patients

To further establish the relationship between AL355338 and ENO1 in NSCLC tissues, we first evaluated ENO1 protein expression with IHC in 80 NSCLC and pair-matched normal tissue samples (Fig. 8A). The results revealed that 76.3% (61/80) NSCLC samples showed increased ENO1 expression compared with their pair-matched normal tissues (Fig. 8B). ENO1 protein levels positively correlated with AL355338 levels (r = 0.382, P < 0.001, Fig. 8C), suggesting potential regulation of ENO1 by AL355338 in clinical NSCLC tissues. Meanwhile, TCGA datasets revealed that ENO1 expression was significantly upregulated in lung adenocarcinoma and lung squamous carcinoma tissues compared to normal tissues (Fig. 8D, E). Kaplan–Meier analysis of the 80-patient NSCLC cohort showed that high ENO1 expression was associated with poor prognosis (P = 0.009, Fig. 8F). This was validated in the TCGA cohort; survival analyses showed that patients with low ENO1 expression had longer overall survival (P = 0.025) and disease-free survival (P = 0.044) compared to those with high ENO1 expression (Fig. 8G, H).

## Discussion

Despite developments in therapeutic approaches, the prognosis for NSCLC patients diagnosed in an advanced stage remains poor [25]. The enhanced Warburg effect is a leading cause of malignant features in NSCLC. Multiple glycolysis-associated genes have been reported as contributors to cancer progression [26]. Earlier studies indicated that numerous lncRNAs were associated with NSCLC initiation and development of NSCLC [7, 27]. Since ^18^F-FDG uptake can reflect the Warburg effect, we previously subjected high ^18^F-FDG-uptake NSCLC tissues to RNA-seq analysis to identify glycolysis-related lncRNAs in our previous study [16]. AL355338 was a newly identified oncogenic lncRNA among the top-scoring upregulated genes. Clinical analysis showed that AL355338 expression was notably higher in tumor tissues than in corresponding adjacent tissues. Importantly, high AL355338 expression in patients with NSCLC was related to advanced TNM stage and poor clinical outcome. Functionally, the positive regulation of AL355338 in promoting NSCLC proliferation and metastasis was identified in a series of in vitro and in vivo experiments. The observation of high AL355338 expression in glycolytically active tissues led us to investigate whether AL355338 could regulate cancer metabolism. In support of our hypothesis, downregulation of AL355338 dramatically weakened the Warburg effect in NSCLC cells. The results indicate an oncogenic role of AL355338 in regulating malignant phenotype, since abnormal cancer cell metabolism is likely to induce cancer aggressiveness.

In addition to its biological importance, we dissected the mechanisms by which AL355338 mediated glycolysis pathway activation. A cross-linking RNA pull-down/MS strategy was used to detect proteins that directly interacted with AL355338. Among the MS data, the glycolytic enzyme ENO1 piqued our interest. As a well-documented glycolytic enzyme responsible for ATP generation, ENO1 is related to mediate energy metabolism and cancer development [11]. Recent studies reported that ENO1 was correlated with cancer differentiation and progression in several tumor types by interacting with lncRNAs. Hong et al. [12] concluded that lncRNA ENO1-IT1, which was activated by *Fusobacterium nucleatum* abundance, could promote glycolysis and oncogenesis by targeting ENO1 and guiding its histone modification pattern in colorectal cancer. Another group confirmed that lncRNA SNHG18 promoted glioma cell motility by disrupting the nucleocytoplasmic shuttling of ENO1 [28]. Consistent with the previous report, our results suggested that ENO1 was responsible for the oncogenic role of AL355338 in mediating NSCLC progression. ENO1 silencing weakened the exogenous AL355338-induced metabolic shift in NSCLC cells. AL355338 also maintained ENO1 protein stabilization through decreasing ubiquitin-mediated degradation. Notably, we observed simultaneous upregulation and correlation of AL355338 and ENO1 in NSCLC tissues. ENO1 and AL355338 overexpression were strongly associated with poor prognosis in NSCLC patients, suggesting that AL355338 and ENO1 levels may predict patient outcome.

In addition to its canonical roles in glycolysis, accumulating evidence revealed ENO1 as a multifunctional protein involved in regulating several signaling pathways [29]. A recent study reported that ENO1 promoted the self-renewal and malignant phenotype of lung cancer stem cells by affecting the AMPK/mTOR pathway [23]. Fu et al. [24] showed that ENO1 was involved in NSCLC glycolysis, proliferation, and metastasis through the FAK-mediated PI3K/AKT pathway. It is rather remarkable that EGFR is the best-characterized receptor tyrosine kinase and EGFR signaling is among the most dysregulated molecular pathways in NSCLC [30]. Upon ligand binding to EGFR, the AKT signaling pathway will be activated, leading to enhanced glycolysis and oncogenic phenotypes in tumor cells [31]. Given the positive correlation of ENO1 and EGFR expression in NSCLC and their similar functions in AKT pathway regulation, these reports inspired us to explore whether ENO1 might be an interaction partner of EGFR. Notably, a direct interaction between ENO1 and EGFR was observed by reverse co-IP, and native ENO1/EGFR protein complexes were visualized with PLA assays. Moreover, our data clearly showed that ENO1 positively regulated EGFR phosphorylation and thus activated downstream AKT kinase signaling.

Previous studies revealed that lncRNAs might act as a tumorigenic regulator by modulating signaling networks [32]. Apart from directly modulating ENO1 expression, we hypothesized that AL355338 functions through the ENO1-mediated EGFR/AKT pathway in NSCLC cells. As expected, ectopic AL355338 expression could enhance ENO1/EGFR interaction, which subsequently led to higher levels of phosphorylated EGFR and AKT. Opposite results were observed in AL355338-depleted cells. However, ENO1 silencing attenuated the increases in phosphorylation modulated by AL355338 induction. ENO1 overexpression restored the reduction in phosphorylated EGFR and AKT levels induced by AL355338 knockdown. Collectively, these results indicated that AL355338 might regulate the ENO1-mediated EGFR/AKT pathway.

## Conclusions

Our study mainly focused on the glycolysis-associated lncRNA AL355338 in NSCLC. Remarkably, AL355338 was increased in NSCLC tissues, and its levels correlated with clinical outcomes, suggesting it could be an independent biomarker in patients with NSCLC. Further functional assays suggested that AL355338 functions as an oncogene by mediating glycolysis activation and promoting NSCLC progression through selective targeting of ENO1 protein. Additionally, we demonstrated that AL355338 stabilized ENO1 protein to prevent ubiquitin-mediated degradation. AL355338 was also capable of modulating interaction between ENO1 and EGFR, further activating EGFR-AKT signaling. In conclusion, our results suggested that upregulated lncRNA AL355338 might serve as a potential biomarker and therapeutic target in the clinical treatment of NSCLC.

## Supplementary Information

**Additional file 1: Table S1.** Primers used in the paper were listed. **Table S2.** Primary antibodies used in the paper were listed. **Table S3.** Mass spectrometry analysis for RNA pull-down. **Figure S1.** The non-coding nature of AL355338 and its expression is up-regulated in NSCLC. **Figure S2.** AL355338 was up-regulated in NSCLC. **Figure S3.** The expression pattern of AL355338 in NSCLC cell lines and its oncogenic roles in promoting EMT of NSCLC cells. **Figure S4.** AL355338 directly binds with ENO1 protein. **Figure S5.** The relationships between ENO1 and EGFR expression in NSCLC.

## Data Availability

All data generated or analyzed during this study are included in this published article.

## Abbreviations

lncRNA: Long noncoding RNA
NSCLC: Non-small cell lung cancer
EMT: Epithelial-mesenchymal transition
FDG: Fluorodeoxyglucose
TNM: Tumor-node-metastasis staging system
IP: Immunoprecipitation
RIP: RNA immunoprecipitation
FISH: Fluorescence in situ hybridization
IHC: Immunohistochemistry
IF: Immunofluorescence
PLA: Proximity Ligation Assay
ENO1: Enolase 1
HK2: Hexokinase II
PKM2: Pyruvate kinase isozymes M2
LDHA: Lactate dehydrogenase A
EGFR: Epidermal growth factor receptor
AKT: Serine/threonine kinase
TCGA: The cancer genome atlas
