# Supplementary material for: AL355338 acts as an oncogenic lncRNA by interacting with protein ENO1 to regulate EGFR/AKT pathway in NSCLC
Source: Cancer Cell Int. 2021 Oct 9;21:525. doi: 10.1186/s12935-021-02232-z (PMC8502354; doi:10.1186/s12935-021-02232-z)
Supplement: Supplementary file 1 — Additional file 1: Table S1. Primers used in the paper were listed. Table S2. Primary antibodies used in the paper were listed. Table S3. Mass spectrometry analysis for RNA pull-down. Figure S1. The non-coding nature of AL355338 and its expression is up-regulated in NSCLC. Figure S2. AL355338 was up-regulated in NSCLC. Figure S3. The expression pattern of AL355338 in NSCLC cell lines and its oncogenic roles in promoting EMT of NSCLC cells. Figure S4. AL355338 directly binds with ENO1 protein. Figure S5. The relationships between ENO1 and EGFR expression in NSCLC. [file 12935_2021_2232_MOESM1_ESM.docx]

**Additional file 1:**

**Table S1. Primers used in the paper were listed:**

| **Gene** | **Primer** | **Sequence(5′-3′)** |
| --- | --- | --- |
| **Primers for qRT-PCR** | | |
| ACTB | forward | CATGTACGTTGCTATCCAGGC |
|  | reverse | CTCCTTAATGTCACGCACGAT |
| AL355338 | forward | CAGCATGGCCAACATGGTGAAG |
|  | reverse | AGTCTGCTGTGTCCTCAACTTCCTTG |
| ENO1 | forward | AAAGCTGGTGCCGTTGAGAA |
|  | reverse | GGTTGTGGTAAACCTCTGCTC |
| U6 | forward | CTCGCTTCGGCAGCACA |
|  | reverse | CTCAACTGGTGTCGTGGA |
| **Sequences for gene knockdown** | | |
| si-AL355338#1 | forward | CCUGAUUUCUGGCCAAUAATT |
|  | reverse | GGACUAAAGACCGGUUAUUTT |
| si-AL355338#2 | forward | GAGAGAAAGAGGAUUGGAUTT |
|  | reverse | AUCCAAUCCUCUUUCUCUCTT |
| sh-AL355338 | CCGGCCACTGTTGAGGTTGATCTCTCTCGAGAGAGATCAACCTCAACAGTGGTTTTTG | |
| sh-ENO1 | CCGGAATGTCATCAAGGAGAAATATCTCGAGATATTTCTCCTTGATGACATTTTTTTG | |

**Table S2.** **Primary antibodies used in the paper were listed:**

| **Name** | **Company** | **Cat. No.** | **Concentration** |
| --- | --- | --- | --- |
| Anti-ENO1 antibody | Abcam | ab227978 | 1:1000 (WB) |
| Anti-ENO1 antibody | Abcam | ab227978 | 1:500 (IF) |
| Anti-ENO1 antibody | Abcam | ab227978 | 1:2000 (IHC) |
| Anti-ENO1 antibody | Abcam | ab227978 | 1:50 (IP) |
| Anti-EGFR antibody | CST Biologicals | #4267 | 1:1000 (WB) |
| Anti-EGFR antibody | CST Biologicals | #4267 | 1:200 (IF) |
| Anti-EGFR antibody | CST Biologicals | #4267 | 1:50 (IHC) |
| Anti-EGFR antibody | CST Biologicals | #4267 | 1:100 (IP) |
| Anti-E-cadherin antibody | CST Biologicals | #3195 | 1:1000 (WB) |
| Anti-N-cadherin antibody | CST Biologicals | #13116 | 1:1000 (WB) |
| Anti-Vimentin antibody | CST Biologicals | #5741 | 1:1000 (WB) |
| Anti-HK2 antibody | Proteintech | 22029-1-AP | 1:1000 (WB) |
| Anti-LDHA antibody | Proteintech | 19987-1-AP | 1:4000 (WB) |
| Anti-PKM2 antibody | Proteintech | 60268-1-lg | 1:2000 (WB) |
| Anti-p-EGFR antibody | CST Biologicals | #2234 | 1:1000 (WB) |
| Anti-p-AKT antibody | Proteintech | 9272S | 1:1000 (WB) |
| Anti-AKT antibody | Proteintech | 4060S | 1:1000 (WB) |
| Anti-flag antibody | Proteintech | 20543-1-AP | 1:1000 (WB) |
| Anti-HA antibody | Biolegend | 901503 | 1:2000 (WB) |
| Anti-ACTB antibody | Proteintech | 60008-1-lg | 1:10000 (WB) |

**Table S3. Mass spectrometry analysis for RNA pull-down.**

| **Accession** | **Description** | **Score** |
| --- | --- | --- |
| Q32P51 | Heterogeneous nuclear ribonucleoprotein A1-like 2 OS=Homo sapiens OX=9606 GN=HNRNPA1L2 PE=2 SV=2 | 234.45 |
| Q13085 | Acetyl-CoA carboxylase 1 OS=Homo sapiens OX=9606 GN=ACACA PE=1 SV=2 | 178.63 |
| P06733 | Alpha-enolase OS=Homo sapiens GN=ENO1 PE=1 SV=2 | 170.88 |
| Q562R1 | Beta-actin-like protein 2 OS=Homo sapiens OX=9606 GN=ACTBL2 PE=1 SV=2 | 167.59 |
| P62136 | Serine/threonine-protein phosphatase PP1-alpha catalytic subunit OS=Homo sapiens OX=9606 GN=PPP1CA PE=1 SV=1 | 151.92 |
| P62140 | Serine/threonine-protein phosphatase PP1-beta catalytic subunit OS=Homo sapiens OX=9606 GN=PPP1CB PE=1 SV=3 | 146.4 |
| Q9NX05 | Constitutive coactivator of PPAR-gamma-like protein 2 OS=Homo sapiens OX=9606 GN=FAM120C PE=1 SV=3 | 142.7 |
| Q99873 | Protein arginine N-methyltransferase 1 OS=Homo sapiens OX=9606 GN=PRMT1 PE=1 SV=3 | 131.88 |
| A0AV96 | RNA-binding protein 47 OS=Homo sapiens OX=9606 GN=RBM47 PE=1 SV=2 | 122.4 |
| Q6UXN9 | WD repeat-containing protein 82 OS=Homo sapiens OX=9606 GN=WDR82 PE=1 SV=1 | 115.62 |
| Q01105 | Protein SET OS=Homo sapiens OX=9606 GN=SET PE=1 SV=3 | 109.95 |
| P57721 | Poly(rC)-binding protein 3 OS=Homo sapiens OX=9606 GN=PCBP3 PE=2 SV=2 | 109.93 |
| O95994 | Anterior gradient protein 2 homolog OS=Homo sapiens OX=9606 GN=AGR2 PE=1 SV=1 | 109.85 |
| P31948 | Stress-induced-phosphoprotein 1 OS=Homo sapiens OX=9606 GN=STIP1 PE=1 SV=1 | 99.03 |
| Q69YQ0 | Cytospin-A OS=Homo sapiens OX=9606 GN=SPECC1L PE=1 SV=2 | 94.29 |
| P49750 | YLP motif-containing protein 1 OS=Homo sapiens OX=9606 GN=YLPM1 PE=1 SV=4 | 91.05 |
| Q5T9A4 | ATPase family AAA domain-containing protein 3B OS=Homo sapiens OX=9606 GN=ATAD3B PE=1 SV=1 | 90.51 |
| Q9UKA9 | Polypyrimidine tract-binding protein 2 OS=Homo sapiens OX=9606 GN=PTBP2 PE=1 SV=1 | 84.06 |
| P22392 | Nucleoside diphosphate kinase B OS=Homo sapiens OX=9606 GN=NME2 PE=1 SV=1 | 81.07 |
| O00763 | Acetyl-CoA carboxylase 2 OS=Homo sapiens OX=9606 GN=ACACB PE=1 SV=3 | 77.66 |

**Additional file 2:**

**
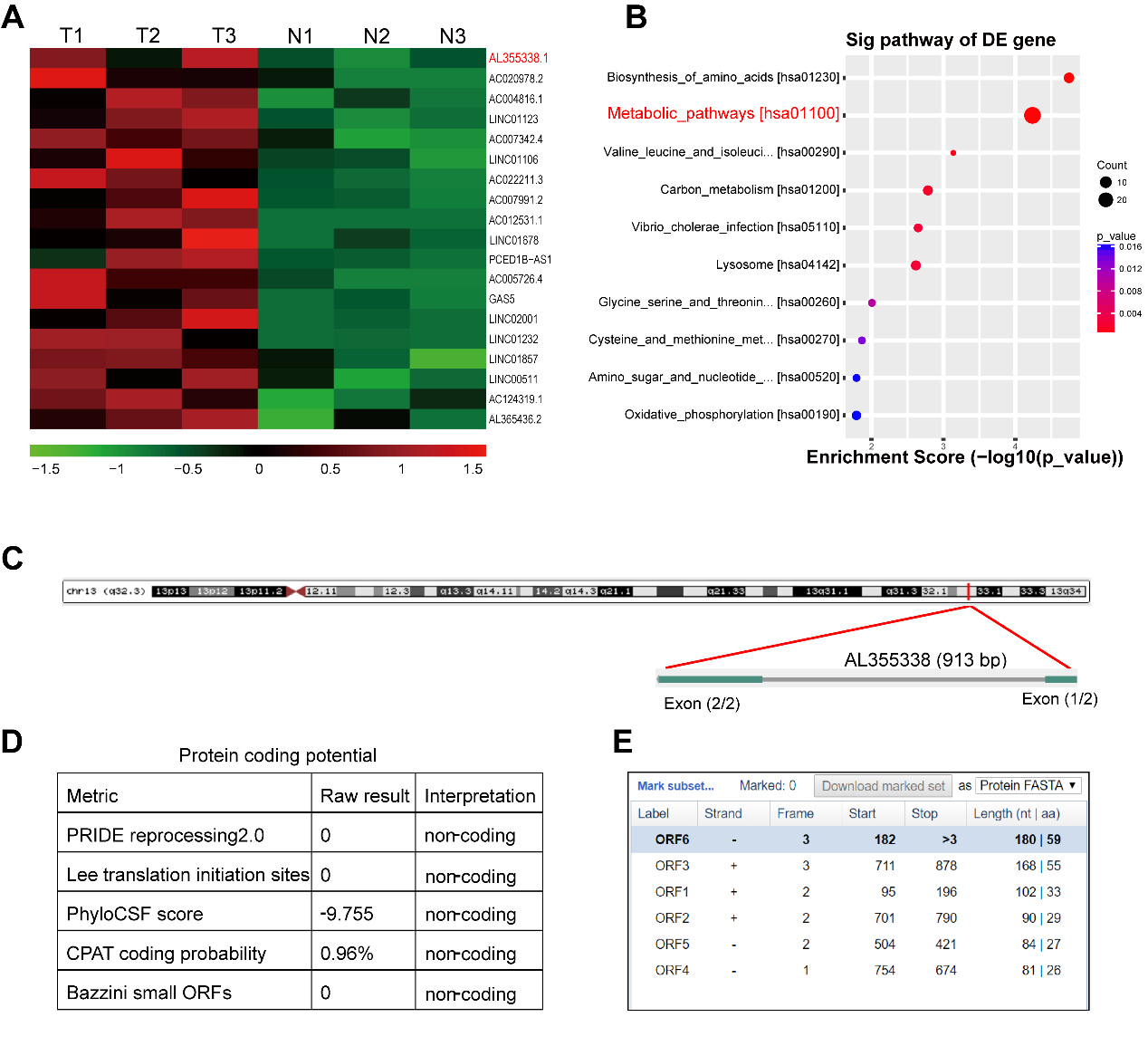
**

**Figure S1. The non-coding nature of AL355338 and its expression is up-regulated in NSCLC.**

(A) The heat map reflects the top 20 upregulated lncRNAs in RNA-seq analysis of 3 paired NSCLC tumor and adjacent tissues. (B) GO analysis showing that the top 20 overexpressed lncRNA were enriched in metabolic pathways. (C) AL355338 is located on chromosome 13 in humans and composed of two exons with a full length of 913 bp. (D) The coding potential of AL355338 in several prediction softwares, and results showed that AL355338 have no coding potential. (E) ORF Finder software prediction (https://www.ncbi.nlm.nih.gov/orffinder/) for the protein-coding potential of AL355338.


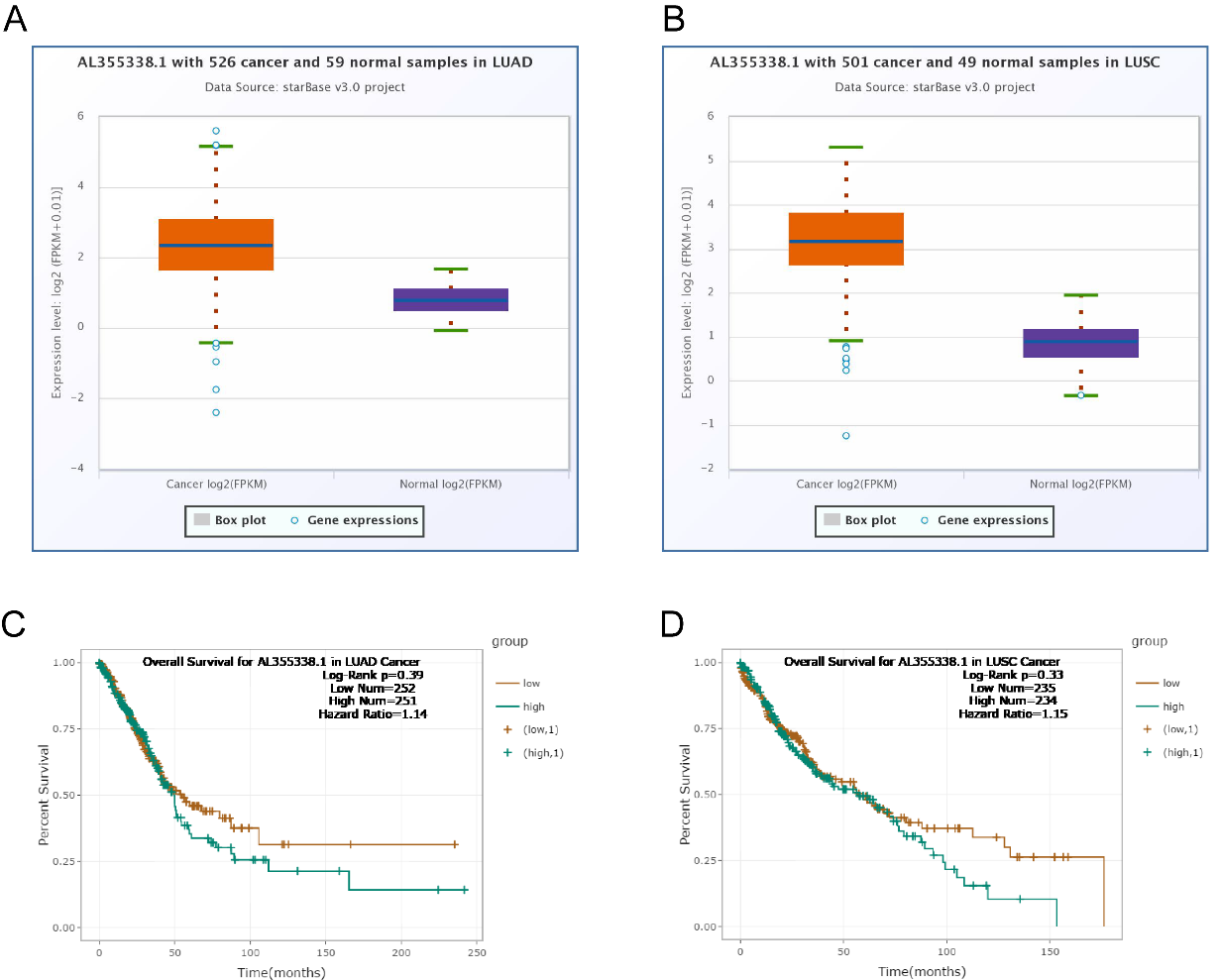


**Figure S2. AL355338 was up-regulated in NSCLC.**

(A) The expression pattern of AL355338 in lung adenocarcinoma based on TCGA data from starBase V3.0 (http://starbase.sysu.edu.cn). (B) The expression pattern of AL355338 in lung squamous carcinoma from starBase V3.0. (C-D) TCGA data from starBase V3.0 Platform further demonstrated that high AL355338 expression indicated poor survival of NSCLC patients. The median expression level of AL355338 was used as the cut-off.

**
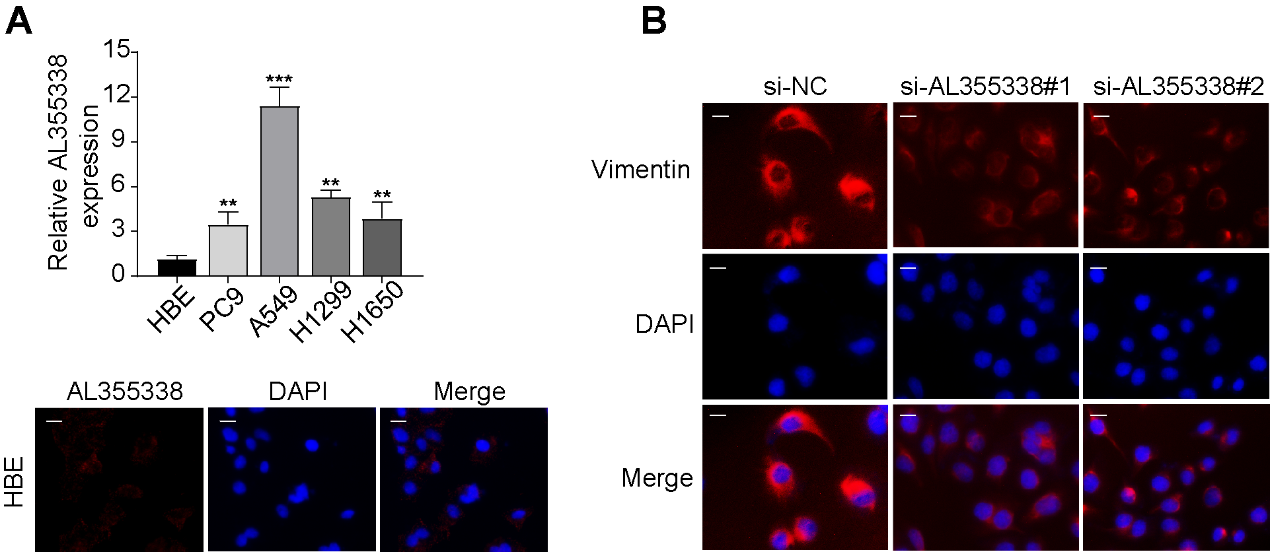
**

**Figure S3. The expression pattern of AL355338 in NSCLC cell lines and its oncogenic roles in promoting EMT of NSCLC cells.**

(A) Expression of AL355338 in four NSCLC cell lines (A549, H1299, H1650, PC9) and the normal lung cell line (HBE) was analyzed by qRT-PCR (in the upper panel). FISH experiments showed that the positive signal of AL355338 expression was extremely low in HBE cells (in the bottom panel). (B) Expression of vimentin was detected by immunofluorescence in A549 cells transfected with negative control group (si-NC), AL355338 siRNAs group. Data shown are mean±SD (n = 3). Scale bar, 10 μm. (*P < 0.05, **P < 0.01, ***P < 0.001)

**
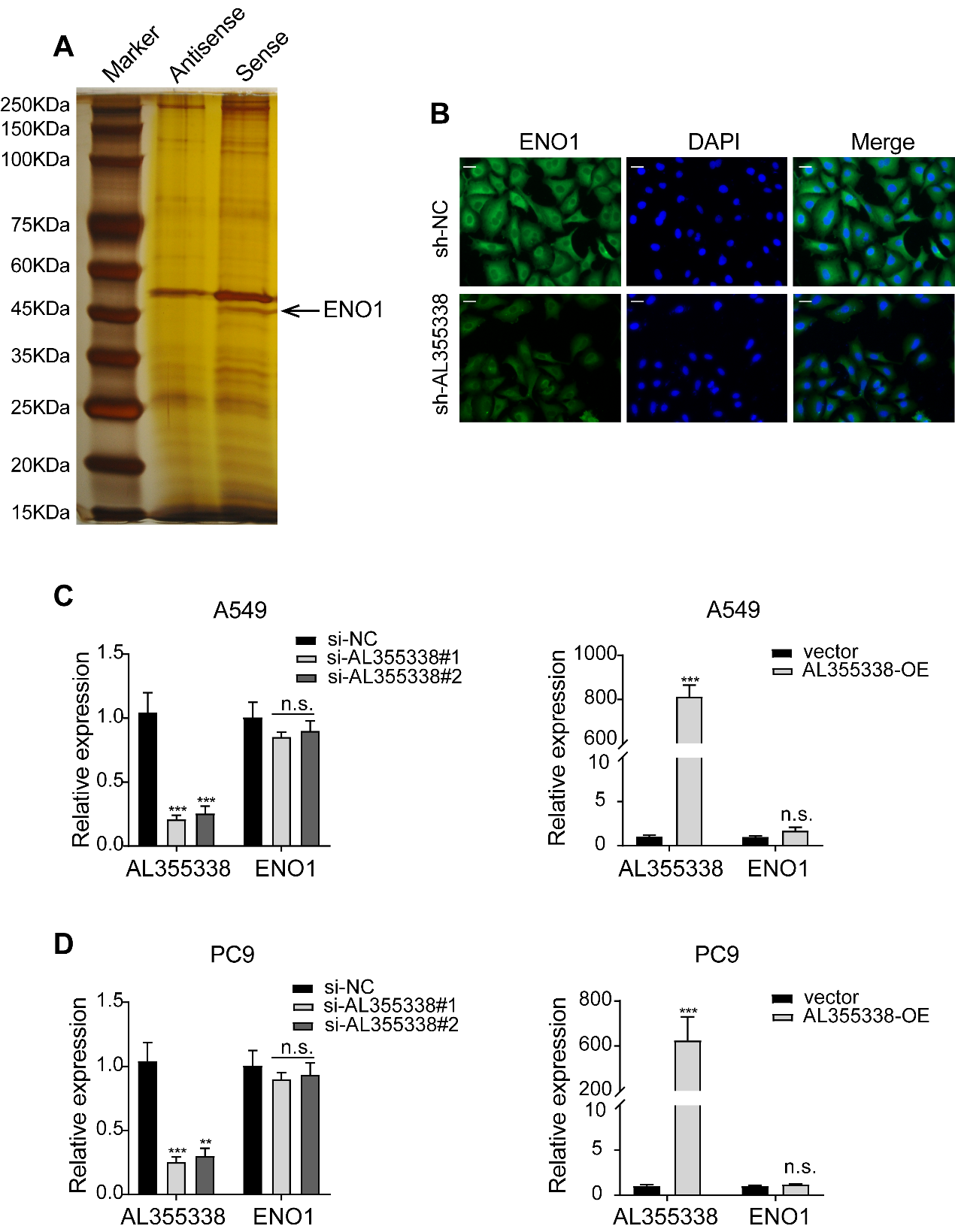
Figure S4. AL355338 directly binds with ENO1 protein.**

(A) Silver-stained SDS-PAGE gel-containing proteins derived from RNA pulldown by AL355338 sense RNA and antisense control RNA. The arrow indicated ENO1 protein from mass spectrometric analysis. (B) Expression of ENO1 was detected by immunofluorescence between sh-AL355338 group and negative control group. Scale bar, 10 μm. (C) ENO1 mRNA expression level was measured by qRT-PCR after knockdown or overexpression of AL355338 in A549 cell. (D) ENO1 mRNA expression level was measured by qRT-PCR after knockdown or overexpression of AL355338 in PC9 cell. Data shown are mean±SD (n = 3). (*P < 0.05, **P < 0.01, ***P < 0.001)

**
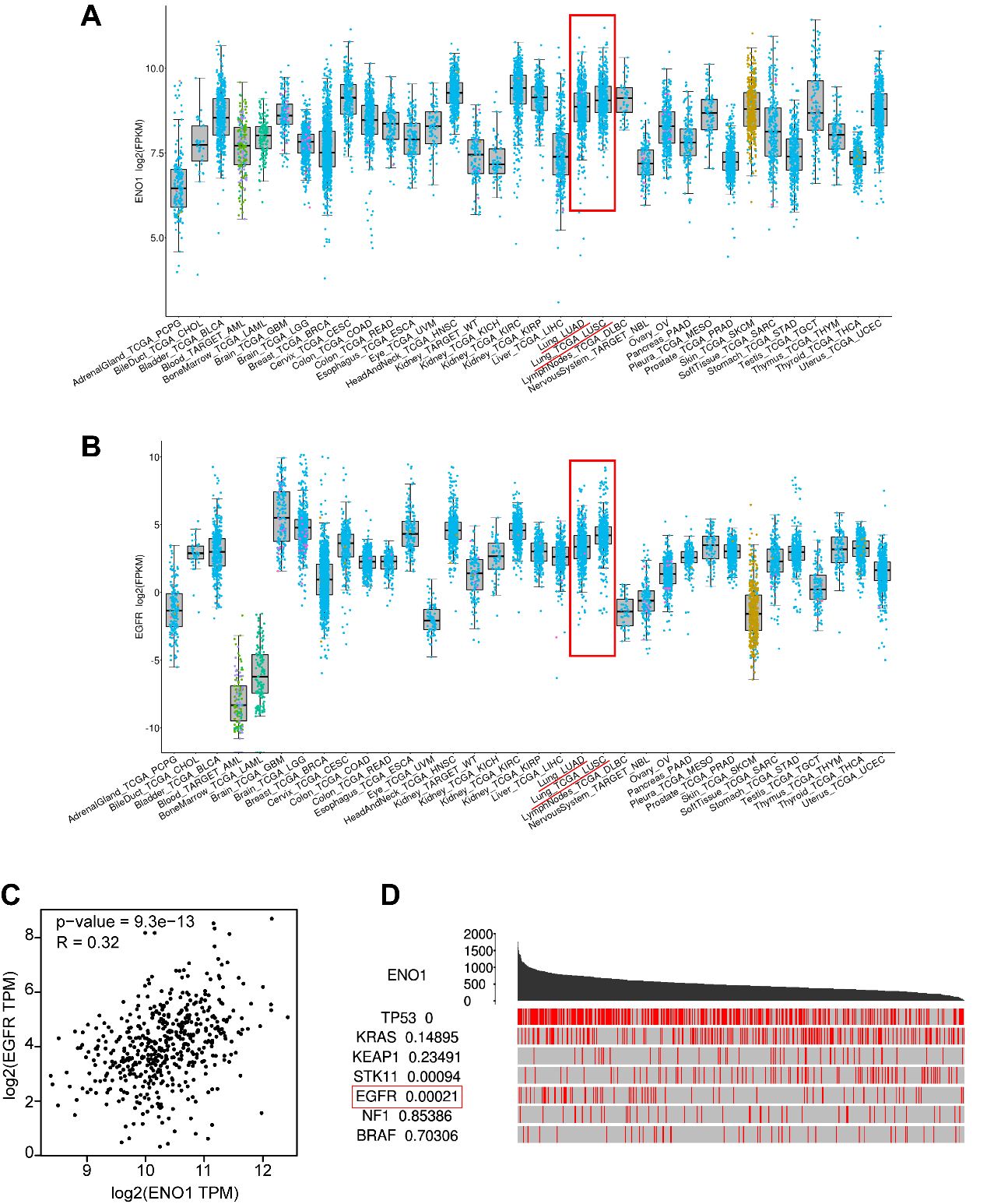
**

**Figure S5. The relationships between ENO1 and EGFR expression in NSCLC.**

(A-B) TCGA datasets from TCGAportal (www.tcgaportal.org): Pan-cancer analysis indicated that ENO1 and EGFR expression was prominently higher across diverse cancer types. (C) A positive correlation between ENO1 and EGFR expression was observed in NSCLC cohort based on TCGA database from GEPIA Platform (http://gepia.cancer-pku.cn/). R = 0.32, P <0.001 by Spearman correlation test. (D) The value of ENO1 mutant status adjacent to highly mutated genes is permutation test p-value of gene expression between driver mutated (red) and not-mutated (gray) samples.
